# The reproduction process of Gram-positive protocells

**DOI:** 10.1038/s41598-024-57369-4

**Published:** 2024-03-25

**Authors:** Dheeraj Kanaparthi, Marko Lampe, Jan-Hagen Krohn, Baoli Zhu, Falk Hildebrand, Thomas Boesen, Andreas Klingl, Prasad Phapale, Tillmann Lueders

**Affiliations:** 1 Department of Cellular and Molecular Biophysics, Max-Planck Institute for Biochemistry, Munich, Germany; 2 Chair of Ecological Microbiology, BayCeer, University of Bayreuth, Bayreuth, Germany; 3 Excellenzcluster Origins, Garching, Germany; 4 Advanced Light Microscopy Facility, European Molecular Biology Laboratory, Heidelberg, Germany; 5 Key Laboratory of Agro-Ecological Processes in Subtropical Regions, CAS, Changsha, China; 6 Quadrum Institute, Norwich, UK; 7 Department of Biosciences, Center for Electromicrobiology, Aarhus, Denmark; 8 Department of Biology, LMU, Planegg-Martinsried, Germany; 9 European Molecular Biology Laboratory, Heidelberg, Germany

**Keywords:** Evolutionary ecology, Microbial ecology, Biogeochemistry

## Abstract

Protocells are believed to have existed on early Earth prior to the emergence of prokaryotes. Due to their rudimentary nature, it is widely accepted that these protocells lacked intracellular mechanisms to regulate their reproduction, thereby relying heavily on environmental conditions. To understand protocell reproduction, we adopted a top–down approach of transforming a Gram-positive bacterium into a lipid-vesicle-like state. In this state, cells lacked intrinsic mechanisms to regulate their morphology or reproduction, resembling theoretical propositions on protocells. Subsequently, we grew these proxy-protocells under the environmental conditions of early Earth to understand their impact on protocell reproduction. Despite the lack of molecular biological coordination, cells in our study underwent reproduction in an organized manner. The method and the efficiency of their reproduction can be explained by an interplay between the physicochemical properties of cell constituents and environmental conditions. While the overall reproductive efficiency in these top-down modified cells was lower than their counterparts with a cell wall, the process always resulted in viable daughter cells. Given the simplicity and suitability of this reproduction method to early Earth environmental conditions, we propose that primitive protocells likely reproduced by a process like the one we described below.

## Introduction

Life on Earth is thought to have emerged in a series of steps described by the theory of chemical evolution^1^. According to this theory, organic compounds essential for building a cell were synthesized by Miller-Urey-like processes^2^ and subsequent prebiotic chemical reactions^3–5^. These organic compounds were thought to have self-assembled into lipid vesicles with intracellular chemical reactions^5^. Eventually, these vesicles evolved in complexity into protocells. These protocells are considered primitive, largely devoid of complex intracellular processes^6,7^. Unlike present-day cells, whose physiology, growth, and reproduction are regulated by complex molecular biological processes^8,9^, such processes in protocells are thought to have been governed by the physicochemical properties of their cell constituents and fluctuating environmental conditions^7,10^. Despite being a critical evolutionary link between abiotic chemical reactions on early Earth and prokaryotes, the existence of protocells and mechanisms of their reproduction largely remained theoretical. Moreover, their reproduction process and the influence of environmental conditions have never been empirically studied. To understand protocell reproduction, we used a top-down approach to transforming a Gram-positive bacterium into a primitive lipid vesicle-like state. We then attempted to understand their reproductive mechanism within their native habitat (i.e., environmental conditions of early Earth).

At present, most, if not all, studies focused on understanding protocells use lipid vesicles (LV) prepared under well-defined laboratory conditions as model protocells^7,11,12^. Our study differs from these previous studies in two key aspects. First, rather than the conventional choice of using LVs, here we used protoplasts of the Gram-positive bacterium *Exiguobacterium* strain-Molly (*EM-P*) as proxy-protocells. Despite accomplishing considerable progress in our theoretical understanding, we believe that studies conducted on LVs do not represent the true complexities of a self-replicating protocell. Even a primitive protocell should have possessed minimal biosynthetic pathways, a physiological mechanism of harvesting free energy from the surrounding (energy-yielding pathways), and a means of replicating its genetic material and transferring it to the daughter cells. These minimal physiological processes could incorporate considerable cytoplasmic complexity. Hence, the biophysical properties of the protocell cytoplasm could have resembled those of the cytoplasm of protoplasts more than an aqueous interior of the LVs.

Protoplasts exhibit several key similarities to protocells, such as their inherent inability to regulate their morphology or reproduction^13,14^. Despite possessing all the necessary genetic information, protoplasts undergo reproduction through simple physiochemical processes independent of canonical molecular biological processes^14^. This method of reproduction is considered to have been erratic and rather primitive^13,15^, akin to the theoretical propositions on protocells^7^. Although protoplasts are fully evolved cells with considerable physiological complexity, the above-mentioned similarities suggest that protoplast reproduction could be similar to that of protocell reproduction^15^. Hence, we propose that protoplasts are a better model system for studying protocell reproduction than LVs.

As a second innovation in our study, we conducted all our experiments under prebiotically plausible environmental conditions to understand how protocells behave in their natural habitat. Given the lack of intracellular complexity, theoretical propositions suggest that environmental conditions likely played an important role in facilitating protocell reproduction^7,10^. In tune with this proposition, studies have shown that the behavior of LVs, when used as model protocells, was considerably influenced by environmental conditions like osmolarity, the nature of salts in the media, and the mechanical stresses^7,10,16^. Despite this theoretical significance of environmental conditions, most current studies on protocells were conducted under standard laboratory conditions rather than probable environmental conditions of early Earth.

The exact environmental conditions of early Earth are a matter of debate among researchers. Nevertheless, an emerging consensus among paleo-geologists suggests that surface temperatures of Archaean Earth (4000–2500 million years ago) ranged between 26° and 35°C^17,18^.The Archaean Eon oceans were also thought to have been 1.5 to 2 times saltier than the current oceans^19^. Moreover, all the earliest known signs of life, like the oldest known microfossils, are restricted to the coastal marine environment and are often encrusted in salt^20,21^. Although paleontological studies do not provide an indication of where life could have evolved, they suggest that protocells/proto life (if not the very first cells) flourished in moderately halophilic environments^22^. To replicate these conditions, we grew the bacterial protoplasts in a medium containing 7% Dead Sea salt (DSS) at 30 °C. DSS was used in our incubations rather than pure NaCl to replicate the complex salt composition of the Archaean oceans^23,24^. In the sections of the manuscript below, we present the lifecycle of these proxy-protocells in their native environment.

## Results

### Transformation of *EM* into its protoplast state (*EM-P*)

**Figure 1:**
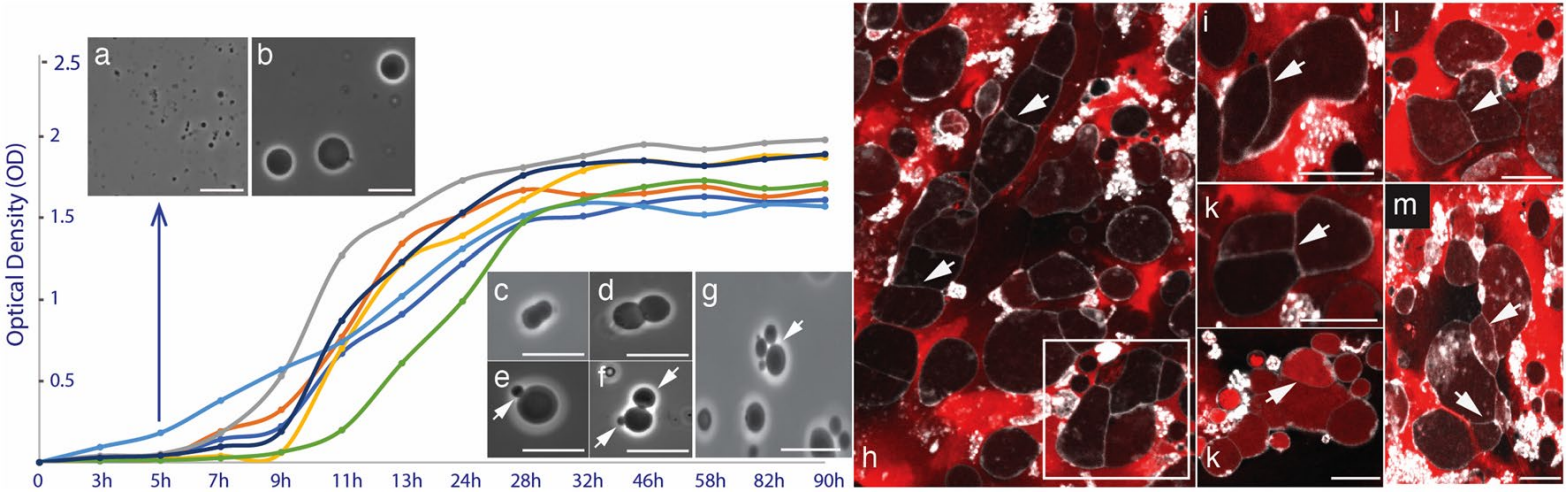
Growth characteristics of *EM-P* in batch culture. *EM-P* exhibited a typical sigmoid growth curve with an initial phase of slow growth rate (lag-phase), a log phase with exponentially increasing cell numbers, and a gradual decline in the growth rate during the stationary phase. The X and Y-axis in the plot indicate the time in hours (h) and optical density (OD). Images (**a,b**) show a comparison of sizes between the cells in the lag and early log phases of growth. Images (**c–g**) show log-phase cells that seem to reproduce either by binary fission ((**c,d**), and Video S1–S3) or budding ((**e–g**), arrows) (Fig. S4). Images (**f–k**) show log phase *EM-P* cells transferred from 7%DSS-TSB to isotonic media rich in organic carbon. Cells in these images were stained with universal membrane stain, FM^TM^ 5–95 (white), and DNA stain, PicoGreen™ (red). Arrows in the images point to membrane septa formed at random places, ultimately resulting in the compartmentalization and formation of individual daughter cells. Scale bars: 5 μm.

*Exiguobacterium strain Molly* (*EM*) was transformed into protoplasts, as described in the methods section. This transformation of “wild-type” *Exiguobacterium strain Molly* (*EM*) into its protoplast state (*EM-P*) was evident from the change in morphology from bacillus to significantly larger spherical cells (Fig. 1). The diameter of these cells ranged between 0.5 and 20 μm. Despite numerous repetitions, no uniformity was observed in the sizes of the cells (Fig. S1). Transferring or culturing cells in media containing the action of specific proteins involved in canonical cell replication, like 3-methoxybenzamide, did not affect the growth characteristics or the morphology of the cells (Fig. S2). Also, in accordance with the previous studies, we observed cells in their protoplast state were in a state of lipid excess (Fig. S3). Most *EM-P* cells had intracellular lipid droplets or hollow filamentous extensions, suggesting excessive lipid synthesis in protoplasts (Fig. S3).

### The life cycle of *EM-P* in 7%DSS-TSB

During the lag and early-log growth phase, *EM-P* gradually grew by an order of magnitude in diameter (Fig. 1a, b, Fig. S1 & Video S1). Subsequently, these spherical cells transformed from spherical to ovoid shapes (Fig. 1c, Video S2). These cells gradually transformed into a dumbbell shape with two distinct daughter cells connected by a membrane tether (Videos S2, S3). These cells subsequently underwent fission, resulting in individual daughter cells. Despite having the appearance of binary fission, no uniformity was observed in the sizes of the daughter cells (Fig. S4). Depending on the size of the daughter cells, cells in this growth stage appear to be reproducing either by budding (Fig. 1b–g, Fig. S4) or by binary fission (Fig. 1e–g, Fig. S4, Videos S2 and S3).

The majority of cells in this growth stage exhibited continuous surface undulations, as seen in Videos S1–S3. Treatment of cells with bactericidal compounds like glutaraldehyde and formaldehyde killed the cells, which is evident from the lack of growth when subculture into new media but did not result in a static cell state. A static state without surface undulations was only observed when the cells were treated with detergents like 0.5% Triton X100 or when transferred to media isotonic to 7%DSS-TSB but devoid of salt and rich in organic carbon (methods section). This transfer of cells into organic carbon-rich media also transformed cells into non-spherical morphologies (Fig. 1h–m). *EM-P* in organic carbon-rich media did not exhibit any distinct method of reproduction resembling budding or binary fission (Fig. 1h–m). Instead, *EM-P* reproduced by an entirely random process. Transfer of cells back and forth from 7%DSS-TSB to organic carbon-rich media resulted in reversible changes in the cell morphology.

**Figure 2:**
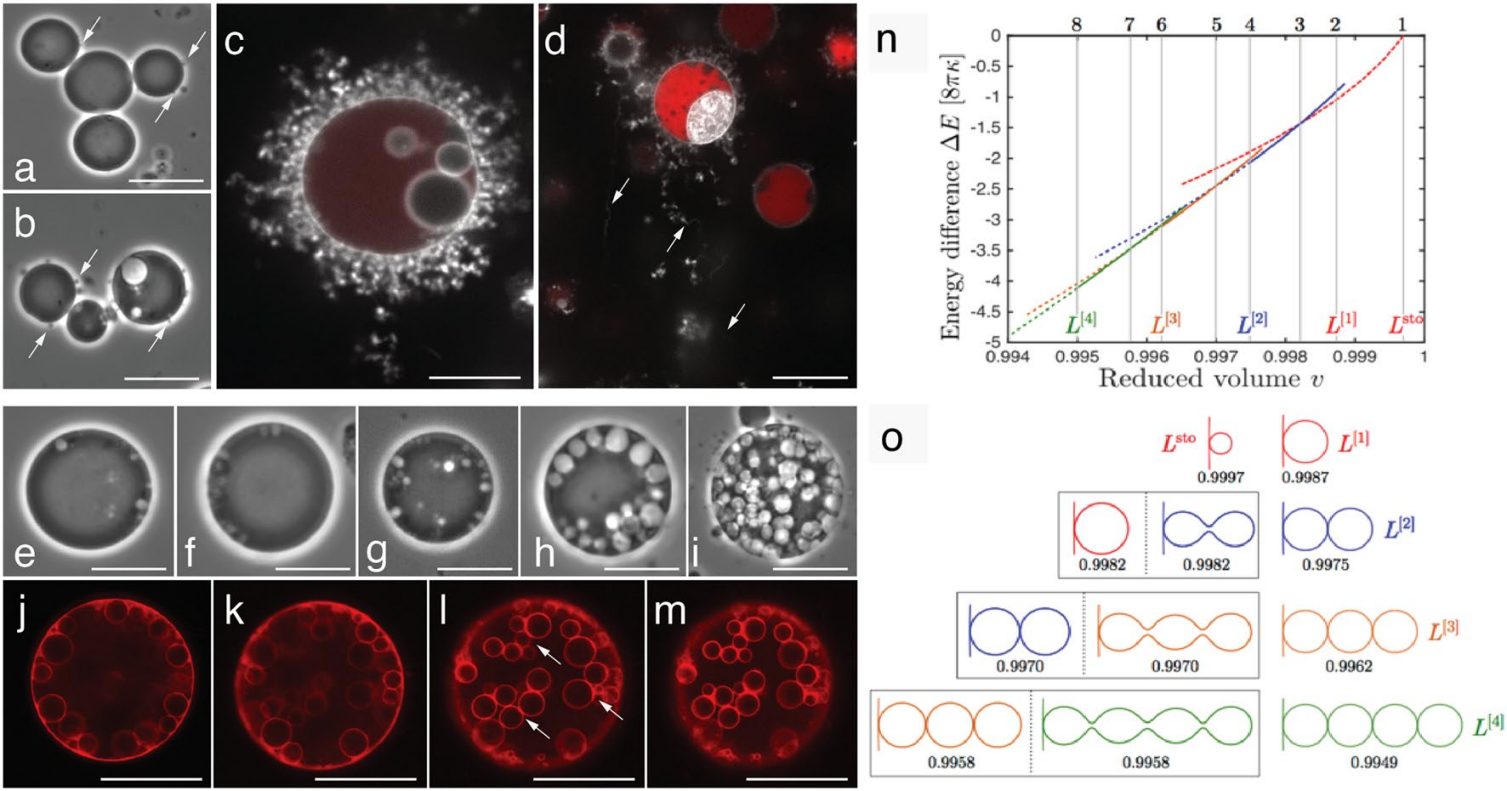
Comparison between theoretically predicted steps in filament formation to observation in *EM-P*. Images (**a–m**) show images of *EM-P* cells from different growth stages forming hollow external filamentous extensions ((**b–d**), arrows) or hollow internal vesicles (**e–m**) in a theoretically predicted pattern (**n,o**). Image (**c**) shows the cell surface covered with short filamentous extensions. Image (**d**) shows cells transferred from 7%DSS to 15%DSS-TSB with longer filamentous extensions. Cells in these images were stained with universal membrane stain, FM^TM^5–95 (white), and DNA stain, PicoGreen™ (red). Images e-i show different stages of intracellular vesicle formation). Images j-m show the formation of a string of hollow spherical vesicles (arrows in these images point to the interconnected nature of these vesicles, Fig. S7), similar to L^(3)^ and L^(4)^ in image-o. Images n & o were originally published by Liu et al*.* 2015 ^25^. Image-n shows the energy landscape of different equilibrium shapes as a function of reduced volume (v). ΔE indicates the deflation-induced (or excess membrane-induced) reduction in the membrane bending energy. Image-o shows the shapes of filamentous extensions corresponding to eight vertical lines in image-n. (please refer to Fig. 5 in Liu et al*.,* 2015^25^ for further information). Scale bars: 10 μm.

Towards the late logarithmic growth phase, we observed cells forming filamentous extensions and hollow intracellular vesicles (Fig. 2). However, we did not observe the formation of these structures when the cells were transferred to fresh media every few hours to keep them in a prolonged logarithmic growth phase. Cells in these incubations continued to reproduce by budding and binary fission. On the other hand, increasing the osmolarity of the media by increasing the salt concentration from 7%DSS to 15%DSS resulted in the osmotic shrinkage of log phase cells and the formation of filamentous extensions (Fig. 2d, Fig. S5). A decrease in the osmolarity of the media by dilution with TSB increased the cell diameter (Fig. S6). We observed a reversible appearance and disappearance of filamentous extensions with an increase and decrease in the osmolarity of the media.

In the subsequent growth stages, we observed *EM-P* reproducing by two different processes—either by forming external or internal daughter cells. Sequential steps involved in reproduction by the formation of external and internal daughter cells are shown in Videos S4–S11 and Videos S12–S16, respectively.

**Figure 3:**
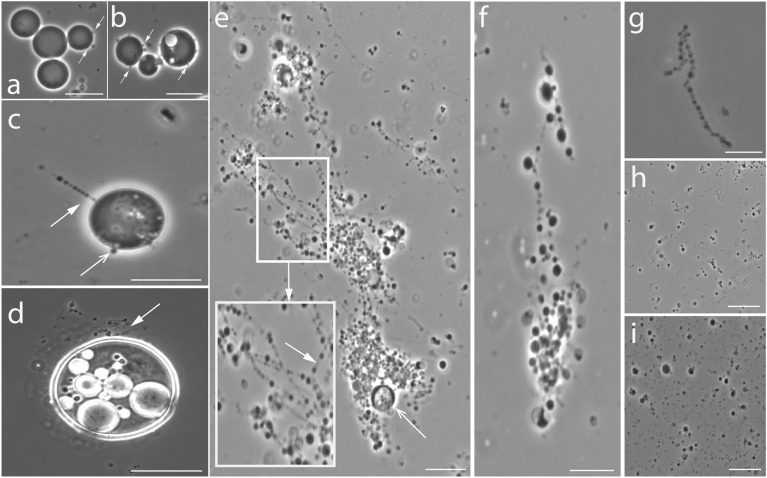
The life cycle of *EM-P* reproducing via the formation of extracellular daughter cells. Images (**a,b**) show a late log phase cell with bud-like structures on the *EM-P* cell surface. Image (**c**) shows a late log phase cell with an open arrow pointing to surface buds and a closed arrow pointing toward filamentous extensions. Image d shows a cell from a subsequent growth stage with multiple filamentous extensions (see Videos S5). Image (**e**) shows the formation of daughter cells as “string-of-beads” attached to a parent *EM-P* cell (open arrow) (also see Videos S6, S7). The magnified region shows the daughter cells formed along the length of these filamentous extensions. The arrow within the magnified region points to the membrane tether connecting daughter cells. Images (**f–i**) show the detachment of these strings of daughter cells and their subsequent fragmentation into individual daughter cells (also see Videos S9–S11). Scale bars: 10 μm (**a–i**).

The formation of external filamentous extensions during the late log phase happened in a series of steps (Fig. 2). *EM-P* cells first developed hollow bud-like structures from multiple locations on the cell surface (Fig. 2a, b). In the subsequent growth stages, these buds increased in length and transformed into short filamentous extensions, often covering the entire surface of the cell (Figs. 2e, 3c, Video S5). These filamentous extensions were never observed to be static and were in a constant state of motion. Over time, the cytoplasm was transferred from the parent cell into these filaments (Fig. 3c, Video S5–S7). These filamentous extensions then developed surface depressions along their length resembling tubular vesicles subjected to curvature-induced destabilization at their surface (like Rayleigh-Plateau instability) (Video S6–S8). This led to the transformation of the filaments from a tubular into a “string-of-beads” morphology (Fig. 3c–e, Videos S6–S8). These strings of daughter cells are then detached from the parent cell (Video S9) and fragmented into individual daughter cells in a sequence of steps, as shown in Fig. 3f–i (Videos S9–S11).

**Figure 4:**
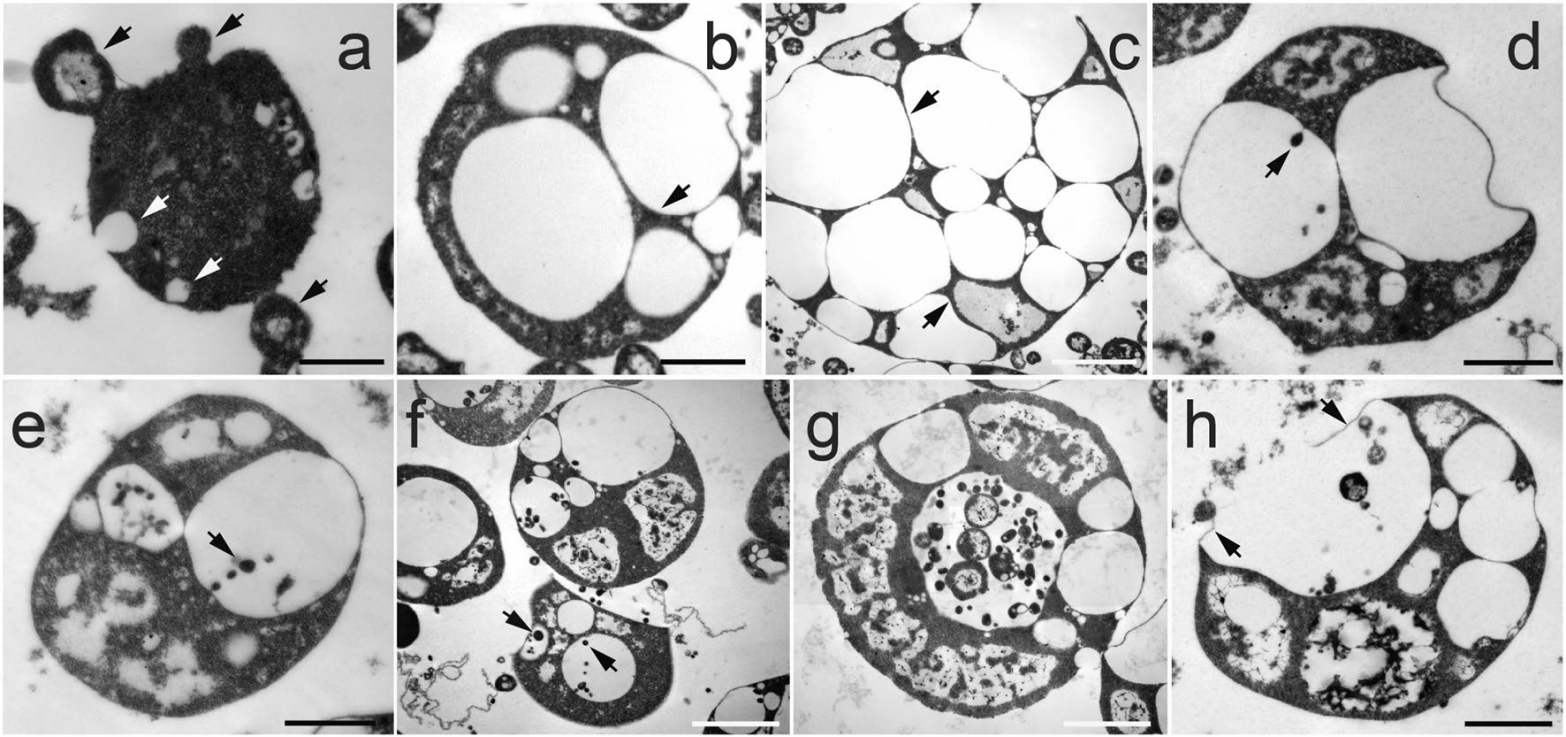
The life cycle of *EM-P* reproducing via the formation of intracellular daughter cells. Images (**a–h**) are TEM images of the life cycle of *EM-P* reproducing by forming intracellular daughter cells. Arrows in images (**b,c**) point to the narrow cytoplasmic region of the cell. Arrows in images (**d,e**) point to the formation of an individual (**d**) and a string of daughter cells (**e**). Image f shows *EM-P* cells with hollow intracellular vesicles with individual daughter cells or a string of daughter cells (Videos S13 and S14). Image (**g**) shows daughter cells of different morphologies within the intracellular vesicles. Image (**h**) shows a rupture of the cell/vesicle membrane (arrows), resulting in the release of the daughter cell (Video S16). Scale bars: 1 μm.

The first step in reproduction via intracellular daughter cells was the formation of hollow intracellular vesicles (Figs. 2e–i, 4). These vesicles were formed by the invagination of the cell membrane by a process resembling endocytosis (Fig. 4a, white arrow). During the late log phase, we observed cells with multiple tiny intracellular vesicles attached to the cell membrane (Fig. 2e–g). Subsequently, these vesicles grew in size (Fig. 2h), detached from the membrane, and migrated into the cytoplasm (Fig. 2i–m, Fig. S7). Often, we also observed these tiny intracellular vesicles being transformed into a string of hollow intracellular vesicles (Fig. 2j–m and Fig. S7). No uniformity was observed in the size or the number of the intracellular vesicles with an *EM-P* cell (Fig. S1). The sizes of these hollow intracellular vesicles ranged between 0.5 and 10 μm.

Daughter cells were formed into these hollow intracellular vesicles by a process resembling budding (Fig. 4d, arrow). The daughter cells are either formed as tiny individual buds attached to the vesicle membrane or as a string of daughter cells originating from different points on the vesicle’s internal surface (Video S13). These buds either detached from the vesicle surface and released into the vesicle as individual daughter cells (Fig. 4f–h, Video S14) or grew into a string of daughter cells (Fig. 4e, arrow, Video S14) and then fragmented into multiple daughter cells (Fig. 4g, h). Daughter cells formed by this process were highly mobile (Video S14). Due to the gradual transfer of cytoplasm from parent to daughter cells (Fig. 4d, e), the cytoplasmic content of the *EM-P* parent cells gradually depleted (Fig. S8). During the late stationary growth phase, many *EM-P* cells were hollow with intracellular vesicles (Fig. 4g, Fig. S8) containing highly motile daughter cells (video. S14). Daughter cells were released into the surrounding media by rupture of the cell or vesicle membrane (Fig. 4h, Video S16). After 5–8 days, most cells in the incubations were tiny daughter cells, a few large *EM-P* cells with intracellular vesicles, and a considerable amount of membrane debris (Fig. S9).

### Membrane phase-separation in *EM-P*

**Figure 5:**
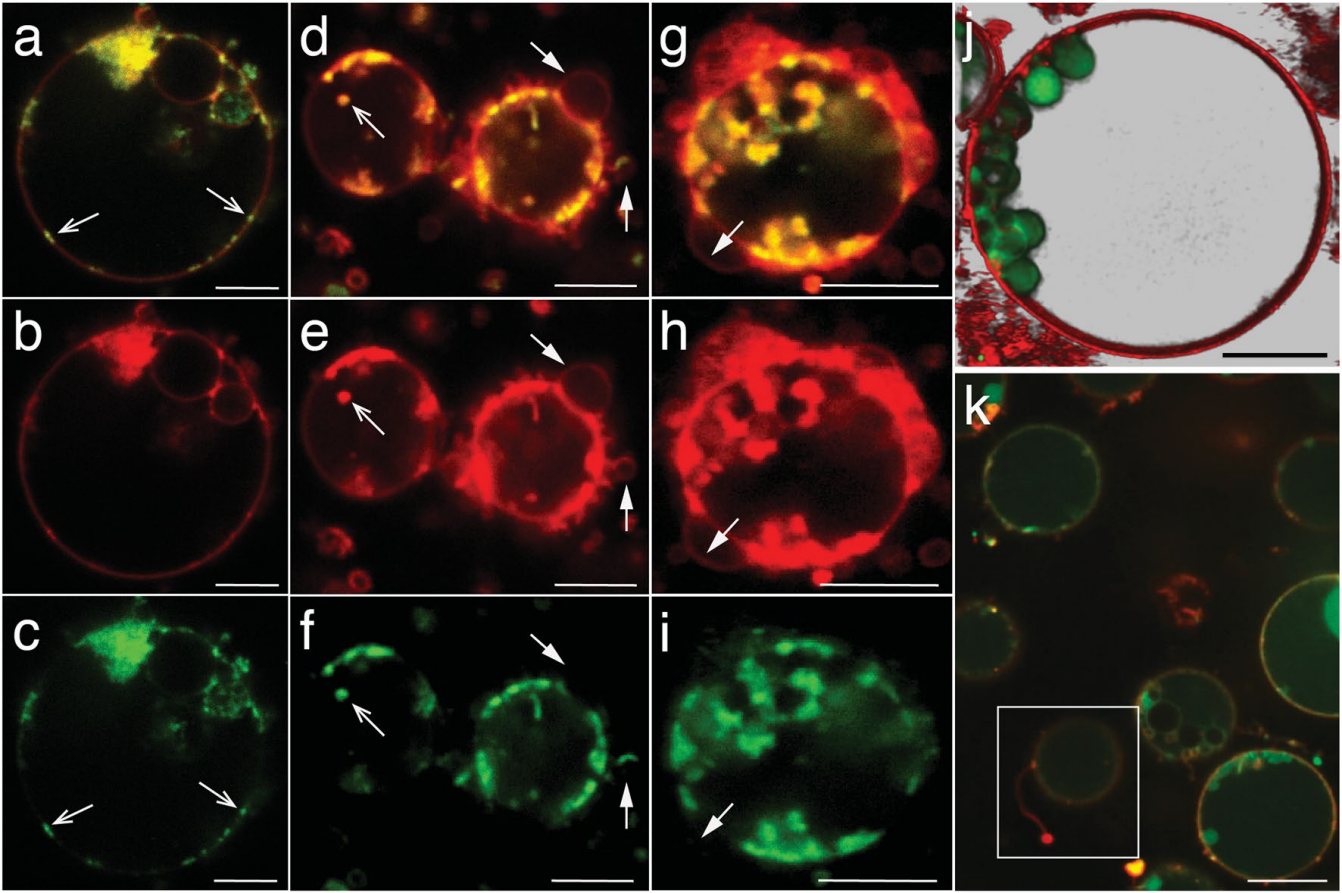
Membrane phase-separation in *EM-P*. Membrane phase-separation in *EM-P*. Cells in these images were stained with FM^TM^5-95 (all membrane, red) and *FAST*™ DiI (L_d_-membrane, green) and imaged either using a super-resolution STED microscope (**a–j**) or a spinning disk confocal (**k**). Images (**a–c,d–f,g–i**) are images of the same cells in different confocal channels. Open arrow in (**a–f**), point to small patches of L_d_-membrane on the cell surface (≈ 180 nm, beyond the diffraction limit of the conventional microscopes) (**a–c**). Closed arrows in (**d–i**) point to L_o_-membrane extensions out of the cell. Image (**j**) is the 3D-rendered image showing *EM-P* cells with intracellular vesicles, that were composed of L_d_-membrane. A series of optical sections of the stacks is shown in Fig. S10. Image (**k**) shows *EM-P* cells with filamentous extensions entirely composed of L_o_ membrane (also see Video S17). Scale bars: 10 μm (**a–i**) and 5 μm (**j,k**).

Controlled splitting (budding and binary fission) and the formation of intracellular vesicles or filamentous extensions in LVs were previously reported to have been associated with membrane phase separation^26^. To observe if such a process is happening in *EM-P,* we stained the cells with dyes specific for the liquid-disordered (L_d_) phase, like FAST-DiI™ and universal membrane dye, FM5-95™. We observed membrane phase separation in *EM-P*’s cell membrane during all growth stages. In early growth stages, L_d_-membrane appeared as small (< 180 nm) patches in the cell membrane, (Fig. 5a–c). The number of these L_d_ patches increased towards later growth stages. Eventually, L_d_-membrane underwent invagination into the cell, leading to the formation of intracellular vesicles (Fig. 5d–i, open arrows). The L_o_-membrane invaginated out of the cell (Fig. 5d–i, closed arrows), ultimately forming filamentous extensions (Fig. 5k and Video S17).

### Influence of environmental conditions on *EM-P*'s life cycle

Environmental conditions strongly influenced the morphology and reproduction process of *EM-P*. Cells grown in 7% DSS-TSB and 7% KCl-TSB were comparable in size and morphology (Fig. S11). In comparison, cells grown in MgCl_2_ (Figs. S2, S13) were significantly smaller than cells grown in equivalent concentrations of DSS or KCl. Apart from the differences in the sizes, a significant portion of cells grown in KCl developed intracellular vesicles with fewer external filaments (Figs. S11, S12). The diameter of most cells grown in MgCl_2_ is restricted to 0.5–2 μm and reproduced mainly by forming extracellular daughter cells (Figs. S11, S12). Also, in contrast to the above-described reproductive process, daughter cells formed by this process did not detach from the parent cell immediately. Over time, they formed an entangled web of cells (Fig. S13b).

When grown on an orbital shaker, *EM-P* reproduced exclusively by binary fission or budding, as shown in Fig. 1a–g. None of the above-described morphologies, like large cells with intracellular vesicles, strings of daughter cells, or membrane debris, was observed. We also observed less membrane debris when grown on an orbital shaker during the stationary phase. Transfer of the culture flasks from an orbital shaker to a static incubator resulted in the appearance of external filamentous extensions and intracellular vesicles. On the other hand, the transfer of cells with filamentous extensions and intracellular vesicles from static incubations to an orbital shaker resulted in cells reproducing by binary fission.

### Efficiency of *EM-P*’s reproduction

**Figure 6:**
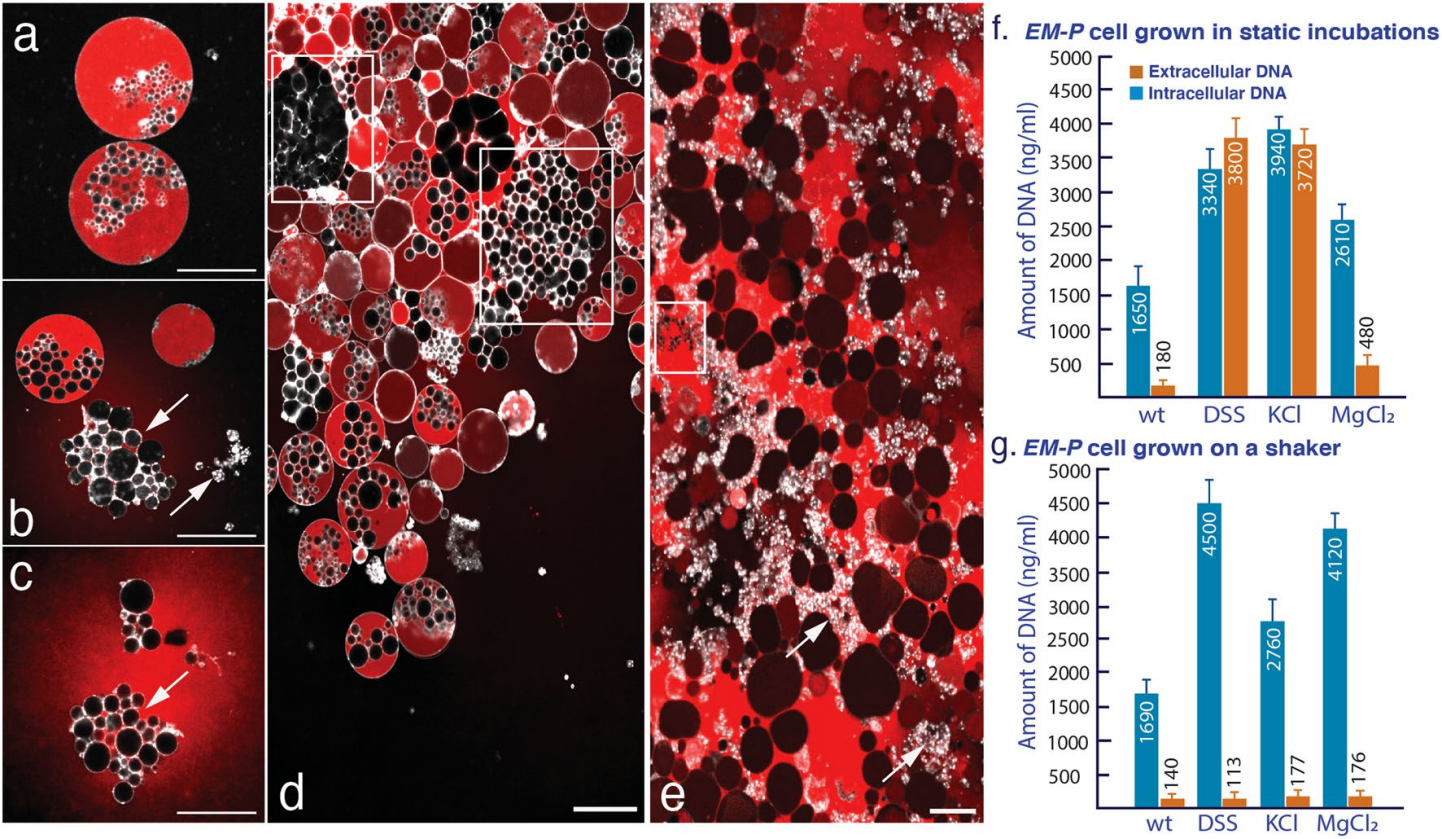
Leakage of cell constituents during *EM-P*’s reproduction. Image (**a–c**) shows the lysis of *EM-P* cells and the release of intracellular DNA (red). Cells in these images were stained with FM^TM^5-95 (membrane, white) and PicoGreen™ (DNA, red) and imaged using a STED microscope. Image d shows a similar process in the biofilm from the static incubations. Highlighted regions in this image show hollow intracellular vesicles released by the lysis of *EM-P* cells. Image e shows late log-phase *EM-P* cells with a considerable amount of extracellular DNA, hollow intracellular vesicles (boxed regions), and membrane debris (arrows). Scale bars: 10 μm. Images (**f,g**) show the results of the quantification of DNA leakage during *EM-P’s* late log growth stage (Fig. S14, for all growth stages) grown under static conditions and on an orbital shaker, respectively. Wt in the plot denotes the wild type (*Exiguobacterium strain M* with a cell wall); DSS, KCl & MgCl_2_ in the plot denoted *EM-P* grown in TSB amended with 7% of the respective salts. Data in images f & g represent mean values of biological repetitions from different batches of *EM-P*’s inoculum (n = 5).

The loss of intracellular DNA during cell division was used as a proxy to determine the reproductive efficiency of *EM-P* (Fig. 6a). The intracellular and extracellular DNA concentrations during all growth stages are shown in Fig. 6f, g, Fig. S14. Given the nature of *EM-P* reproduction (lysis and release of intracellular daughter cells—Fig. 4h, Video S16), we expected some loss of intracellular constituents. However, we also observed spontaneous lysis of cells during all growth stages that is not associated with the release of daughter cells (Videos S21, S22).

**Figure 7:**
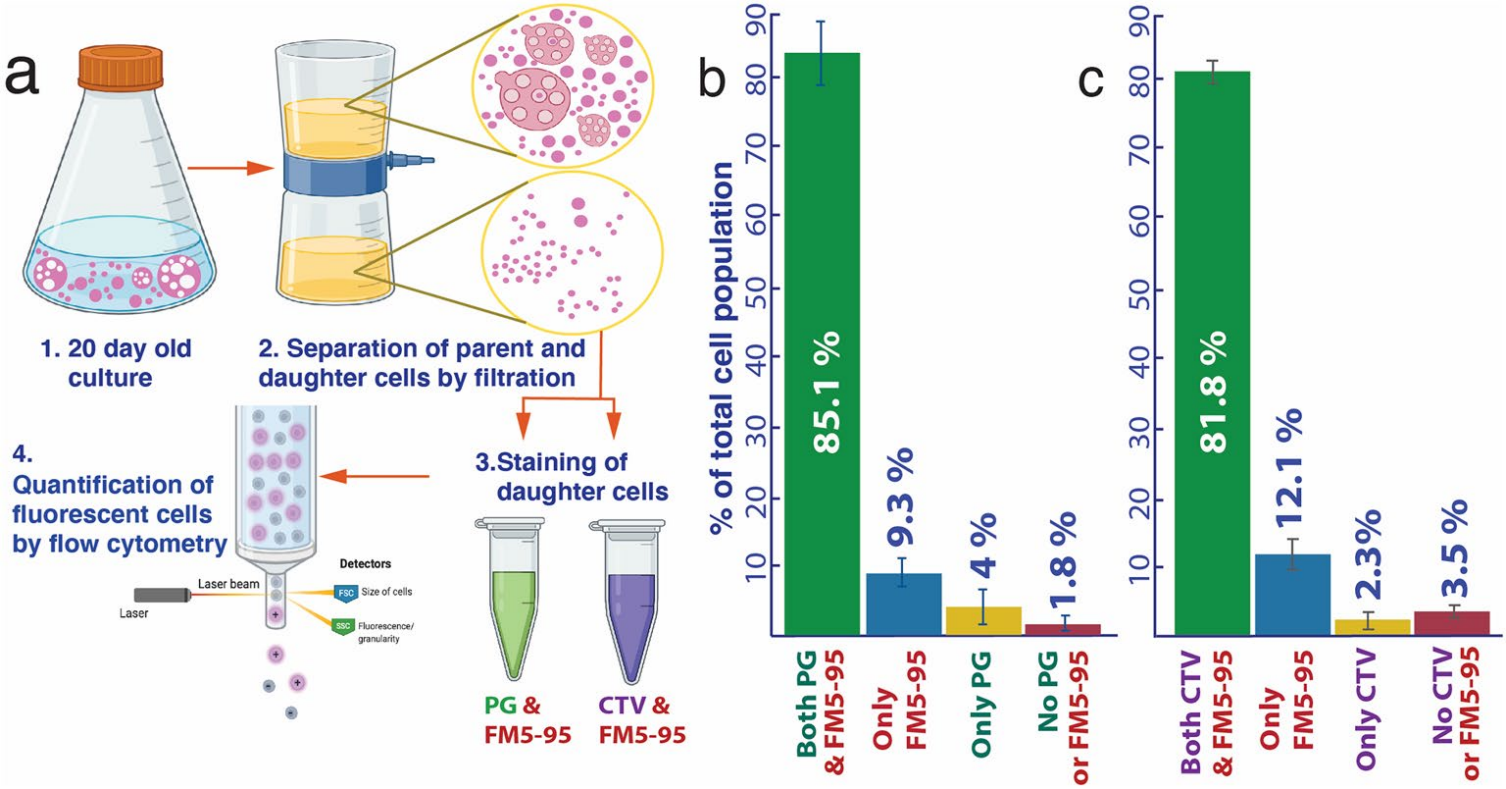
Metabolic viability of daughter cells. (**a**) The workflow of the experiment. Image (**b**) shows the results of the quantification of cells with intact membrane (FM^TM^5–95) and intracellular DNA (PicoGreen-PG) by flow cytometry (the green bar). The subsequent blue, yellow, and red bars show the percentages of cells that were either deficient in intracellular DNA, an intact membrane, or both. Image-c shows the results of the quantification of cells with an intact membrane (FM^TM^5–95) and intracellular enzyme activity (CTV-CellTrace™ Violet) by flow cytometry (green bar). The subsequent blue, yellow, and red bars show the percentages of cells deficient in intracellular enzyme activity, an intact membrane, or both. The original flow cytometry plots, together with the confocal microscope images of daughter cells, were shown in Figs. S15 and S16.

Compared to *EM*, all *EM-P* incubations exhibited a higher concentration of extracellular DNA when grown under station conditions (Fig. 7, Fig. S14). A similar trend was observed in the intracellular and extracellular DNA ratio when *EM-P* was grown in media containing DSS or KCl (Fig. 7, Fig. S14). In comparison, less leakage of DNA was observed when cells were grown in media containing MgCl_2_. In contrast to the static incubations, significantly less leakage of DNA was observed when *EM-P* was grown on an orbital shaker (Fig. 7, Fig. S14). No significant differences were observed between the native *EM* and *EM-P* in terms of DNA leakage when *EM-P* cells were grown in media containing MgCl_2_ on an orbital shaker.

### Viability and metabolic activity of *EM-P* daughter cells

The viability of daughter cells formed by the above process was determined by separating daughter cells by filtration and inoculating them into fresh 7%DSS-TSB media. All such incubations always resulted in growth. The percentage of viable daughter cells was determined by the staining of the daughter cells for the intracellular DNA (Fig. 7b), cytoplasmic enzyme activity (Fig. 7c), and subsequent quantification of cells by flow cytometry. Our results suggest that Ca. 80 to 85% of the daughter cells receive DNA from the parent cells and exhibit cytoplasmic enzyme activity (Fig. 7, Figs. S15, S16). The filtrate containing the daughter cells also exhibited good growth when inoculated into 7%DSS-minimal salt media ^27^ containing glucose, ribose, and sucrose as the sole carbon and energy source (Fig. S17).

## Discussion

Due to the absence of a cell wall, protoplasts exhibit random morphologies and reproduce in a disorganized manner^13^. This method of reproduction is shown to have been independent of canonical molecular biological processes that typically govern bacterial reproduction^14^. Beyond disruption to their morphology and reproduction, recent studies demonstrated that the loss of cell wall has more profound implications on cell physiology. Several studies reported discrepancies in intracellular functions, like an uncontrolled synthesis of cell wall precursors, even among stable protoplasts that lack a cell wall^28^, the inability to maintain a proper intracellular oxidative state^29^, uncontrolled replication of the genome^30^, and excessive lipid synthesis^31^. These studies suggest that protoplasts lack coordination among different physiological pathways within the cell.

A similar lack of coordination among intracellular pathways was also observed in *EM-P*. Cells in our experiments were in a perpetual state of lipid (membrane) excess (Fig. S3). Moreover, *EM-P* showed no difference in their morphology or reproduction process in the presence or absence of cell-division inhibitors (Fig. S2). In tune with the previous studies^14,31^, these observations suggest a lack of intracellular coordination among intracellular processes, and canonical molecular biological processes did not play a role in determining cell division in *EM-P*. However, in sharp contrast to the earlier studies^13^, this loss of intracellular coordination did not result in an entirely random reproduction in *EM-P*. Under the environmental conditions of early Earth, *EM-P* still reproduced in a defined sequence of steps, always leading to the formation of viable daughter cells. Rather than intracellular processes, this method of reproduction can be explained by the biophysical properties of its cell constituents, such as the growth rate, excessive lipid synthesis, and environmental conditions.

**Figure 8:**
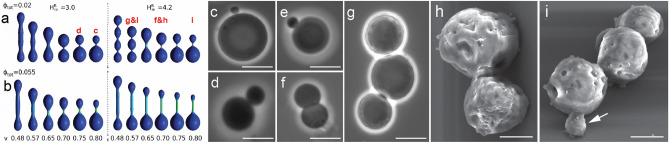
Asymmetric division of *EM-P* cells. Images (**a,b**) show theoretically predicted morphologies of vesicles with lateral membrane heterogeneity (originally published by Bobrovska et al., 2013)^32^. v is the ratio between the actual volume of a vesicle to the volume of a vesicle in its spherical form, H^B^_m_ is the bending rigidity of the anisotropic component (lipid-lipid or lipid-protein nanodomains within the membrane), and Ф_tot_ is the concentration of the membrane component that differed in its intrinsic membrane curvature. Images (**c–i**) show phase-contrast (**c–e**) and SEM (**h,i**) images of *EM-P* cells undergoing a similar method of reproduction. Similarities between *EM-P* cells and predicted morphologies are indicated in the image (**a**) (letters in red). Scale bars: 10 μm (**c–e**) & 2 μm (**h,i**).

The sequential transformation of *EM-P* observed in our study closely resembles that of LVs undergoing osmotic shrinkage^25,33^ (Fig. 8a, b). Due to the presence of excess membrane during osmotic shrinkage, LVs are known to transform into a variety of non-spherical morphologies^32,34^. The morphology of these LVs is dependent on the V. The V or “the reduced volume” of an LV is defined as the ratio of the actual volume of the cell (V) to its volume in its spherical form (V_s_), i.e., V = V/V_s_. A spherical cell with no excess membrane has a V of 1, given the spherical form has the lowest volume-to-surface-area ratio ^32^. However, if the membrane surface area increases disproportionate to its volume (like in the case of osmotic shrinkage), it leads to high V-values (V > 1). This results in the transformation of spherical LVs into various non-spherical morphologies. Like the LVs, morphologies observed in *EM-P* (Fig. 8), can be explained by the differences in V during different growth stages.

During the early growth stages, *EM-P* cells increased in size but retained their spherical morphologies (Fig. 1). Like in LVs, the spherical morphology of *EM-P* cells can be explained by the high growth rate of cells in their logarithmic growth phase. Given this rapid growth, we presume that the cytoplasmic volume increase (i.e., growth rate) kept pace with excessive lipid synthesis, resulting only in a minor mismatch between the cell volume and surface area of the cell membrane, i.e., V = 1. A gradual reduction in the growth rate of *EM-P* due to the depletion of nutrients in the media likely results in a mismatch between the rate of increase in the cell volume and the membrane surface area, resulting in higher V. As predicted by the theoretical models^34,35^, this increase in the V could have led spherical *EM-P* cells to transform into ovoid and then to dumbbell morphologies, ultimately resulting in the formation of two distinct daughter cells (Videos S1–S3).

The heterogenicity in the sizes of daughter cells (Fig. 1, Fig. S4) can be attributed to the complex chemical composition of cell membranes. Most previous studies attempting to understand the biophysical properties of protocells were conducted on LVs with simple chemical composition (two to three components). Due to the equilibration of these constituents within the membrane, the physical properties, like elasticity and spontaneous curvature, remain uniform along their surface. In contrast to LVs, cell membranes are composed of numerous lipid species, quinones, membrane proteins, and, in the case of *EM-P,* a diverse composition of carotenoid pigments^36^. This diverse composition results in the formation of lipid-lipid^37^ and lipid-protein^38^ nanodomains with different physical properties^32^ along the surface of the cell membrane, resulting in spatial differences in the membrane elasticity and bending rigidity. Such multicomponent membranes with lateral heterogenicity (designated by H^B^_m_) are theoretically predicted to undergo budding and formation of different daughter cells of different sizes^39^ (Fig. 2).

An even further increase in V due to a gradual reduction of growth rate and the presence of excess membrane resulted in the formation of hollow intracellular vesicles and filamentous extensions as a mechanism for accommodating this excess membrane. Similar behavior of LVs has been reported previously in LVs^25,40,41^. In line with our proposition that growth rate determined the morphology of *EM-P*, cells kept in a continuous log phase did not develop filamentous extensions or intracellular vesicles. The formation of such structures in log-phase *EM-P* cells could only be induced by a sudden increase in the osmolarity of the media (Fig. S5). Like in late log phase cells, this increase in the osmolarity resulted in high V due to the shrinkage of the cells.

The sequential stages of filamentous extensions and hollow intracellular vesicle formation in *EM-P*, also resemble the process previously reported in LVs^25,40^. The formation of tiny hollow buds from multiple sites on the LV surface was shown to happen during the slight osmotic shrinkage of vesicles (Fig. 2). On the contrary, the formation of long filamentous extensions and intracellular vesicles was observed during the rapid shrinkage of vesicles by a sudden increase in the osmolarity of the media. The formation of these two distinct structures in LVs is explained by the rate of increase in the membrane surface area—a slow increase is shown to thermodynamically favor the formation of buds from different locations on the LV surface, while a sudden availability of excess membrane thermodynamically favors the elongation of buds into filamentous extensions rather than the formation of new buds^25,40^.

Like in LVs, the sequential stages of the formation of these structures in *EM-P* can be explained by the mismatch between the cytoplasmic volume and the amount of excess membrane. The nearly spherical morphologies of log phase cells suggest a close match between the cell volume and surface area of the cell membrane, i.e., less membrane was available for forming hollow filamentous extensions. This led to the slow transfer of lipids from the cytoplasm to the membrane, forming tiny hollow membrane buds from multiple sites on the cell surface (Fig. 2). The reduced growth rate and excessive lipids in the stationary phase cells could have energetically favored an increase in the length of the existing membrane extensions (internal and external Fig. 2) rather than forming new membrane invaginations on the cell surface. Transfer of late log-phase cells from media with 7%DSS to 15%DSS to create a sudden state of lipid excess led to osmotic shrinkage of cells and abrupt increase in the membrane area. In accordance with previous observations on LVs^25^, these cells were observed to have formed longer and fewer filamentous extensions (Video S6, Fig. 2d) without the initial appearance of multiple membrane buds on the cell membrane.

The formation of external and inter-membrane structures can be explained by the model proposed by Lipowsky and Jülicher^42,43^. It was proposed that phase-separated membranes tend to undergo budding to minimize interfacial energy at the phase boundary to attain a thermodynamically stable state. They do so by forming an out-of-plane membrane invagination, which gradually transforms the softer liquid-disordered (L_d_) membrane into a bud (Fig. 5). Earlier studies hypothesized that the L_d_ membrane most likely undergoes budding due to its low bending rigidities and high spontaneous curvature^25^. In accordance with this theory^37^, budding in *EM-P* is associated with phase separation of the cell membrane (Fig. 5). L_d_ membrane initially appeared as small patches on the cell surface, less than 180 nm in diameter. As predicted by the theoretical propositions, as the area of the L_d_ membrane increased, it underwent budding, possibly due to a gradual increase in the line tension at the phase-boundary^44^ (Fig. 5 , Video S19). Nevertheless, some deviations from this expected behavior were observed. First, despite its higher bending rigidity, the liquid-ordered (L_o_) membrane also underwent budding. Second, was the difference in the direction of budding. Contrary to the predictions, we observed that the L_d_-membrane consistently underwent a negative (into the cell) rather than positive (out of the cell) curvature (Fig. 5). Intriguingly*,* all positive (out of the cell) membrane invaginations were exclusively composed of L_o_-membrane. All negative membrane invaginations (into the cell) were exclusively composed of L_d_-membrane (Fig. 5, Fig. S10, Video S17).

The direction and physical nature of a membrane undergoing budding could be explained by its lipid composition^39^, and the nature of the aqueous core^45^. Bacia et al*.* studied the influence of membrane sterol composition on budding^39^ and reported that the nature and direction of budding is influenced by the nature and concentration of sterols in the membrane. Despite higher bending rigidities, this study showed that some sterols could induce preferential budding of more rigid L_o_ membrane instead of L_d_ membrane. Prokaryotes are known to lack sterols like cholesterol and its derivatives but are known to possess functional analogs of cholesterol, like hopanoids^46^. The properties of these compounds in inducing membrane phase separation and budding were rarely investigated. Apart from sterols, invagination of a more rigid L_o_ membrane was also reported to happen when LVs^47^ experienced a state of excess membrane (like during the osmotic shrinkage), possibly due to an increase in the spontaneous curvature of the L_o_ membrane due to excess lipids^25^.

The nature and direction of the membrane invagination could also have been determined by the packing densities of the aqueous core (cytoplasm) and the growth media surrounding the cell^45^. Studies showed that when liquid–liquid phase separation was induced in the aqueous core (not the membrane), the vesicle membrane also underwent phase separation according to their densities^25,40^. The denser phase of the aqueous core was observed to have been associated with the L_d_ membrane and the lighter phase with the L_o_ membrane^45^. This could explain the invagination of the L_d_ membrane into the cell, given the higher densities of cytoplasm (~ 17 to 35 wt% molecules). Preference of the L_o_ membrane to the comparatively less dense growth media (~ 6 to 7 wt% molecules) could have led to the L_o_ membrane undergoing positive curvature, i.e., out of the cell (Fig. 5 & Video S17). TEM images of cells shown in Fig. 4 show these differences in the packing densities between the cytoplasm and surroundings^48^.

One key aspect of the cell cycle is the fission of the parent cell into metabolically viable daughter cells^49^. Archiving cell division without the aid of molecular biological processes has been the primary focus of multiple studies and is of crucial importance in enhancing our understanding of most primitive cell replication processes ^50^ and building autonomously replicating synthetic cells^49,51^. Previous studies archived fission either by mechanical means ^52^, by encapsulating protein involved in cell division, or by an uncontrolled splitting of LVs by gradually increasing the size of the LVs to the point of instability^7^. Given that *EM-P* was grown under static conditions in the form of a biofilm, we do not presume that the physical stimulus required for splitting the parent cell into two daughter cells (binary fission) originated outside the cell. We rather propose that the force required for splitting the cell originated within the cell and likely was a result of interactions between the intracellular constituents and the cell membrane.

The log phase *EM-P* cells were observed to be in a constant state of surface undulations (Video S1–S3). A similar phenomenon of surface instability ultimately leading to cell fission has been theoretically predicted in liquid droplets with internal chemical reactions^54^ and experimentally demonstrated in LVs-packed proteins known to mediate bacterial cell division^53^. Surface deformations observed in both these cases are attributed to the Brownian movement of the intracellular molecules. This constant movement of intracellular constituents is thought to have provided the kinetic energy required to split the cell into two daughter cells ^54^. Consistent with such studies, we observed intracellular constituents within *EM-P* (like the DNA), in a constant state of movement (Video S20). In a normal bacterial cell, DNA forms highly organized and condensed into a structure like a nucleoid or attached to the cell membrane via a cation-mediated salt-bridge^55,56^. The formation of DNA-DNA and DNA-membrane complexes is known to have been mediated by divalent cations, which neutralize negative charges of the phosphate groups^57^ and form a salt bridge between the phosphate groups of the membrane and DNA. In the case of *EM-P*, no such organizational structure was observed. DNA is distributed evenly throughout the cytoplasm as loose strands (Video S20). This suggests minimal interaction between individual DNA strands or between DNA and the cell membrane. The lack of these interactions could have led to surface undulations due to the electrostatic repulsion between the negatively charged phosphate groups of DNA and the membrane. This constant repulsion and movement of intracellular constituents could have provided the kinetic energy required for cell fission.

Apart from the Brownian movement of intracellular constituents, surface undulations could also be attributed to the transient binding of cations onto the membrane surface^58^, the differences in the osmolarities on either side of the membrane, and compositional differences in the nature of osmolytes on either side of the membrane^33,58^. In the case of *EM-P*, the osmolarity of the cytoplasm was a result of high packing densities of intracellular constituents (organic in nature)^33^, while the osmolarity of the surrounding media was primarily a result of inorganic salts (7%DSS) in the growth media. To understand if these differences played a role in causing the surface undulations, we transferred cells from 7%DSS-TSB into an isotonic growth media containing organic carbon. The absence of cell membrane undulations in these incubations strongly suggests that the nature of the osmolytes in the growth media likely played a role in inducing surface undulations. Apart from not observing surface deformations, cells in organic carbon-rich media cells exhibited completely random morphologies and did not reproduce by the above-described process. The transfer of cells back and forth between the salt-rich and organic carbon-rich media changed their morphologies reversibly. These results suggest that environmental conditions not only determined the morphology of *EM-P* cells but also facilitated cell division.

We do not presume cell division in *EM-P* was a result of specific intracellular protein^53^, as treatment of cells with bactericidal chemicals like sodium azide (0.3%)^59^ or protein polymerizing agents like 0.5% glutaraldehyde and formaldehyde^60^ did not halt the surface undulations in the cell surface. These surface undulations or movement of cells could only be halted by osmotically equilibrating cytoplasm with the surrounding (e.g., by treating cells with Triton X100). In tune with the above results, this suggests that these surface deformations could have been caused either by the differences in osmolarities between the cytoplasm and the surrounding media^61^ or due to the Brownian movement of intracellular constituents ^54^, rather than the action of specific proteins within the cell.

Differences observed in the cell sizes, when grown in the presence of monovalent and divalent salts, can be explained by the interactions between different species of cations and phospholipid head groups^33,62,63^. Interaction of monovalent cations like potassium is known to increase the membrane's fluidity, whereas divalent cations like magnesium decrease the fluidity^62^. This increase in membrane fluidity could have facilitated the expansion of cells in incubations containing DSS and KCl^33^, which could have resulted in relatively larger cells. Increased membrane rigidity when grown in the presence of MgCl_2_ could have led to the formation of smaller cells (Fig. S12). *EM-P* reversibly changes its morphology when transferred between media of different salt compositions. This suggests that the chemical composition of the growth media (i.e., environmental conditions), rather than the information encoded in its genome, influenced the morphology of *EM-P*.

The reasons for the reproduction predominantly via extracellular daughter in the presence of MgCl_2_ and predominantly via intracellular daughter cells in the presence of KCl are currently unclear. Nevertheless, a similar phenomenon of intracellular vesicle formation in the presence of KCl was observed previously in LVs^33^. Apart from the visible differences in morphologies, *EM-P* reproduced more efficiently when grown in the presence of MgCl_2_, compared to DSS or KCl (Fig. 6, Fig. S14). This improved efficiency could be attributed to reproduction by forming external daughter cells in MgCl_2_, which could have eliminated the need for cell lysis to release the intracellular daughter cells (Video S16).

Unlike the growth conditions used in our static incubations, cells in natural environments rarely experience static conditions. Archaean Eon oceans are thought to have had a greater intensity of waves than the present-day Earth due to the closer proximity of the moon during the Archaean Eon ^64^. Hence, protocells inhabiting early Earth’s oceans or shorelines could have been subjected to constant agitation due to this wave action. The reproduction efficiency was significantly higher when the cells were grown on an orbital shaker. This was possible due to the reproduction of cells predominantly by budding or binary fission, eliminating the need for lysis and release of the daughter cells. These results suggest that the reproductive efficiency of *EM-P*, like protocells inhabiting early Earth, could have been similar to cells in present-day cells under favorable environmental conditions, even in the absence of dedicated molecular biological processes. Nevertheless, reproductive efficiency observed in cells from this study is considerably lower and involves the loss of a significant amount of intracellular constituents compared to the previous studies on model protocells^7,65^.

Previous studies employing LVs suggest that the presence of two layers of cell membrane improves the efficiency of reproduction due to the initial expansion of the outer membrane, which forms a hollow template for the expansion of the inner membrane, transforming spherical cells into filamentous morphology followed by fragmentation of cells into individual daughter cells^7^. In tune with this theory, Gram-negative protocells reproduced in a similar manner^65^. The absence of such transformation in *EM-P* could be attributed to the single layer of the cell membrane surrounding the cell (Gram-positive). Apart from reproducing in a manner previously reported from LVs (proxy-protocells), multilamellar or proxy-Gram-negative protocells reproduced with better efficiency than the cells from our study^65^.

A recent study reported protoplasts could replicate their genome, in an uncontrolled fashion, resulting in very high chromosome copy numbers^30^. Together with lipid synthesis, this suggests some loss of coordination within other intracellular processes like DNA synthesis and segregation. Despite these disruptions, our data suggest that most daughter cells (75–80%) received DNA from the parent cells (Fig. 7, Figs. S15 and S16). *EM-P* daughter cells exhibited a good growth rate when transferred to 7%DSS-TSB or into 7%DSS-MSM containing only glucose, ribose, or sucrose as a carbon source (Fig. S17). This ability of *EM-P* to satisfy all its anabolic and energy needs from a single organic carbon suggests that daughter cells possess a full complement of genes to support autonomous growth. These results show beyond a reasonable doubt that *EM-P* is capable of transferring a complete copy of its genome and undergoing cell fission even when devoid of a dedicated molecular biological process.

## Conclusion

Our results suggest that reproduction is an intrinsic biophysical property of cells with an internal metabolism. Even in the absence of canonical molecular biological mechanisms that regulate reproduction, cells can still reproduce efficiently. In such cells, environmental conditions play an essential role in determining their method of reproduction and reproductive efficiency. The influence of environmental conditions was also evident from the reversible changes in the morphologies of the cell with changing culturing conditions like the salt content, the nature of the cations in the media, and mechanical stresses experienced by the cells. Such a profound influence of environmental conditions on bacterial cell reproduction has never been reported before. Our study also demonstrates that working with protoplasts rather than lipid vesicles as proxy-protocells and working under natural environmental conditions rather than well-controlled laboratory conditions provides a better understanding of the behavior of protocells. Given the suitability of this reproduction process to the environmental conditions of early Earth and its simplicity, we propose that Gram-positive protocells inhabiting early Earth likely reproduced by a similar process.

## Methods

### Transformation of cells into stable protoplasts state

*Exiguobacterium* strain-Molly (*EM*) was isolated from submerged biofilms in the Dead Sea ^66^. The phylogenetic affiliation of this isolate to the genus *Exiguobacterium* was determined by 16S rRNA gene sequencing ^67^. *EM* cells were transformed into their protoplast state according to the procedure described before ^16^. In brief, cells were transferred to 3× TSB containing 0.3 M sucrose, 0.5% glycerol, and 300 mg of BSA, with Lysozyme (80 µg/ml) and Penicillin G (200 µg/ml) (Sigma Aldrich, Germany). The transformation of *EM* cells into protoplasts (*EM-P*) was confirmed by observing the cells under a microscope (procedure described in sections below). The resulting *EM-P* cells were transferred into half-strength TSB with 7% Dead Sea Salt (7%DSS-TSB) at regular intervals for over a week (media with Penicillin G and Lysozyme). To check the stability of protoplasts, cells were subsequently transferred into 7%DSS-TSB without Lysozyme and Penicillin G. Given that the cells did not revert to their native state in the absence of antibiotic stress, use of lysozyme and penicillin G was discontinued for subsequent experiments.

### Microscopic observation of *EM-P* cells

Morphology *EM-P* was determined at regular intervals using an Axioskop 2plus microscope (Carl Zeiss, Germany) using a Plan-NEOFLUAR 100×/1.3 objective. Images were acquired with a Leica DSF9000 camera (Leica Microsystems, Mannheim, Germany). STED microscopy was performed with an inverted TCS SP8 STED 3× microscope (Leica Microsystems, Mannheim Germany) using an 86×/1.2 NA water immersion objective (Leica HC PL APO CS2—STED White). Fluorophores were excited with 488, 561 nm, 594 nm, or 633 m laser light derived from an 80 MHz pulsed White Light Laser (Leica Microsystems, Mannheim Germany). For stimulated emission, either a pulsed 775 nm laser or a 592 nm CW laser (Leica Microsystems, Mannheim, Germany) was used depending on the fluorophore. The emitted fluorescence light was collected with Hybrid Detectors (HyD, Leica Microsystems, Mannheim Germany) using appropriate emission band pass-filter setting and typically a gate of 0.3–6.0 ns for depletion with the 775 nm laser or 1.0–6.0 ns with the 592 nm laser. Images were recorded in photon counting mode and line accumulation. Image deconvolution was performed on selected images and movies with Huygens Professional (version 16.10.1p2, Scientific Volume Imaging, Hilversum, The Netherlands).

Spinning Disk Microscopy was performed using an Olympus SpinSR10 spinning disk confocal microscope (Olympus, Tokyo, Japan) equipped with a 100×/NA1.35 silicone oil immersion objective (Olympus UPLSAPO100XS, Tokyo, Japan), a CSU-W1-Spinning Disk-Unit (Yokogawa, Tokyo, Japan) and ORCA‐Flash 4.0 V3 Digital CMOS Camera (Hamamatsu, Hamamatsu City, Japan).

### Transmission electron microscopy

Cells at various growth stages were collected from the growth media by centrifugation at 500 rpm for 5 min. Subsequently, the cells were then preserved in 2.5% v/v glutaraldehyde (Carl Roth, Karlsruhe, Germany). Following preservation, cells were stained with 0.25% w/v uranyl acetate solution. The next step involved dehydrating the cells using increasing concentrations of acetone. Afterward, cells were embedded in epoxy resin and left for polymerization for a duration of 72 h. Epoxy blocks were sectioned, and cells were counter-stained with 1% lead citrate solution. Transmission electron microscopy was carried out using a Zeiss EM 912 (Zeiss, Oberkochen) equipped with an integrated OMEGA filter at 80 kilovolts (kV). Image acquisition was performed with 2k ×2k pixel slow-scan CCD camera (TRS, TrÖndle Restlichtverstrkersysteme, Moorenweis, Germany) with ImageSP software (version 1.2.9.77) (SysProg, Minsk, Belarus).

### Influence of intracellular processes on *EM-P*’s morphology and reproduction

To understand whether intracellular processes played a role in regulating *EM-P*’s morphology or reproduction, cells were inoculated into 7% DSS-TSB media, with and without 5 mM 3-methoxybenzamide. 3-methoxybenzamide was known to inhibit FtsZ function ^68^, a protein known to play a central role in mediating bacterial reproduction. The cells in both incubations were observed at regular intervals under a phase-contrast microscope to detect any disparities in morphology between the control and test incubations.

Previous studies reported excessive lipid synthesis in protoplasts. To determine the presence of excessive lipid synthesis in *EM-P*, we stained cells with lipid-specific dyes like Nile red (Invitrogen, Germany) or FM^TM^5-95 (Invitrogen, Germany) and imaged using STED or confocal microscopes as described above.

### The life cycle of *EM-P* in 7%DSS-TSB

To determine the life cycle of *EM-P*, we inoculated cells into fresh 7%DSS-TSB and observed the cells using various microscopic procedures described above. The growth of *EM-P* cells was quantitatively determined by measuring the optical density (OD) at regular intervals using a Biophotometer^®^ D30 (Eppendorf, Germany). Unless otherwise stated, all cultivation flasks were incubated under static conditions at 30 °C. To determine the influence of mechanical forces on *EM-P*’s morphology and reproduction, another set of identical incubations were conducted in which culture flasks were placed on an orbital shaker at 160 rpm.

To understand the influence of the osmolarity of the media on *EM-P*’s morphology, we increase the osmolarity of cells by adding an appropriate amount of 30%DSS-TSB to the original 7%DSS-TSB to bring the final concentration of DSS in the media to 15%. To determine the influence of the nature of the osmolites in the media on cell morphology, we used media with minimal salt but rich in organic carbon. This media is composed of 3X TSB containing 0.3 M sucrose, 0.5% glycerol, 300 mg of BSA, and 20 mg of extracellular DNA.

To determine the cause of the surface undulations in *EM-P*, we treated cells with different bactericidal compounds like sodium azide (0.3%), with 2% methanol-free formaldehyde and 0.5% glutaraldehyde (Sigma Aldrich, Germany), or with membrane pore-forming compounds like 0.3% Tween 100 (Sigma Aldrich, Germany).

### Membrane phase separation

Several morphological features of *EM-P*, like reproduction by budding or binary fission, were known to have been associated with membrane phase separation in LVs ^34^. To understand if a similar process taking place in *EM-P*, we stained the cell membrane with universal membrane dye FM^TM^5-95 (Invitrogen, Germany) and dye specific for L_d_-membrane, *FAST*™ Dil (Invitrogen, Germany). After staining, cells were observed under a STED or a spinning disk microscope, as described above.

### The life cycle of *EM-P* in 7%KCL-TSB and 7%MgCl_2_-TSB

To determine the influence of different cations on the life cycle of *EM-P*, we cultured the cells in 7%KCL-TSB and 7%MgCl_2_-TSB. The microscopic monitoring of the cells and the quantitative determination of growth were conducted as described above. Another set of parallel incubations were conducted by incubating the cells on an orbital shaker (160 rpm) to determine the influence of mechanical forces on *EM-P*’s reproduction. *EM-P* cells were also transferred between media containing different salts (like transferring the cells from 7%DSS-TSB to 7%MgCl_2_-TSB) to understand if morphology is a result of environmental conditions. Similar experiments were conducted by transferring cells from static incubations onto an orbital shaker. The changes in the cell morphology upon the transfer into this new media were monitored using the microscopic techniques described above.

### Determination of reproductive efficiency

Reproductive efficiency was determined by the leakage of intracellular DNA (as a proxy for intracellular constituents) during *EM-P*’s reproduction. To quantify extracellular and intracellular DNA, we inoculated *EM-P* into all the above-described media compositions (7%DSS, 7%MgCl_2_ and 7%KCL) under static conditions and on an orbital shaker at 160 rpm. 2 ml of cells were harvested every day (24 h) from all the incubations. Intact cells were precipitated by centrifugation at 6000 rpm for 10 min. DNA was extracted from the cells according to the procedure described before. DNA was also extracted from the supernatant by precipitation with two times the volume of ice-cold ethanol and subsequent centrifugation for 20 min at 1400 rpm. The pellet was dissolved in 1 ml of TE buffer. The proteins were removed by phenol–chloroform extraction and subsequent proteinase K treatment (Sigma Aldrich, Germany). The resulting DNA was washed twice by precipitation with 70% ethanol and redissolving the pellet in 200 μl TE buffer (Qiagen, Germany). DNA from all the extractions was quantified using the Qubit dsDNA quantification kit (Invitrogen, Germany).

### Determining the metabolic viability of daughter cells

The viability of daughter cells was determined by inoculating 10–20 day-old cultures into fresh media either directly or after passing them through a 0.45 μm cellulose acetate filter to get rid of large parent cells and membrane debris (Schematic version of the procedure shown in Fig. 8). Cytoplasmic activity in daughter cells was tested by staining daughter cells with DNA stain, PicoGreen™, and live cell labeling CellTrace™ violet stain (Invitrogen, Germany) and imaging the cells using a confocal microscope. The relative percentage of cells with and without cytoplasmic activity and intracellular DNA was quantified by Attune CytPix flow cytometer (Thermo Fischer Scientific, Germany).

The metabolic viability of *EM-P* daughter cells was also determined by transferring the filtrate with daughter cells into 7%DSS-minimal salt media ^27^ with 2%w/v of either glucose, sucrose, or ribose as the sole carbon source. The growth of *EM-P* was tested by measuring the OD of the cultures at regular intervals.

### Supplementary Information

Supplementary Information.

Supplementary Video 1.

## Data Availability

All data, materials, and methods will be shared by Dr. Dheeraj Kanaparthi upon reasonable request.
