# Supplementary material for: The reproduction process of Gram-positive protocells
Source: Sci Rep. 2024 Mar 25;14:7075. doi: 10.1038/s41598-024-57369-4 (PMC10963365; doi:10.1038/s41598-024-57369-4)
Supplement: Supplementary file 1 — Supplementary Information. [file 41598_2024_57369_MOESM1_ESM.pdf]

Supplement - The reproduction process of Gram-positive protocells

Dheeraj Kanaparthi[1,2&3*], Marko Lampe[4], Jan-Hagen Krohn[1&3], Baoli Zhu[2&5], Falk Hildebrand[6], Thomas Boesen[7], Andreas Klingl[8], Prasad Phapale[9], & Tillmann Lueders[2*]

Affiliations:

[1] Department of Cellular and Molecular Biophysics, Max-Planck Institute for Biochemistry, Munich, Germany.

[2] Chair of Ecological Microbiology, BayCeer, University of Bayreuth, Germany.

[3] Excellenzcluster ORIGINS, Garching, Germany.

[4] Advanced Light Microscopy Facility, European Molecular Biology Laboratory, Heidelberg, Germany.

[5] Key Laboratory of Agro-ecological Processes in Subtropical Regions, CAS, Changsha, China.

[6] Quadrum Institute, Norwich, UK.

[7] Department of Biosciences, Center for Electromicrobiology, Aarhus, Denmark.

[8] Department of Biology, LMU, Planegg-Martinsried, Germany.

[9] European Molecular Biology Laboratory, Heidelberg, Germany.

* Corresponding author

Supplementary Figures:

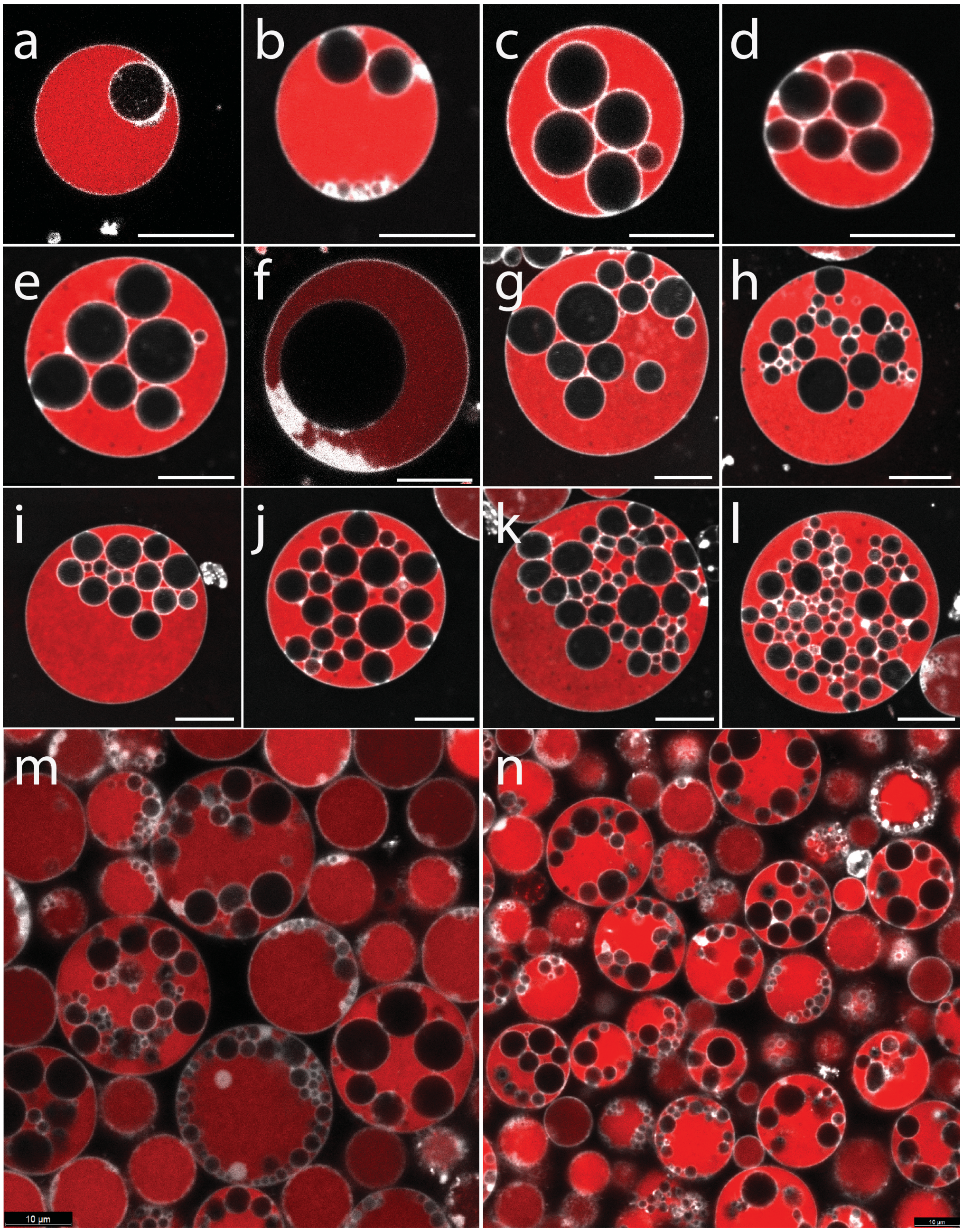

Figure S1: Variation in the size and morphology of *EM-P* cells.

Images a-l are STED microscope images of *EM-P* cells. These images show variations in cell sizes, size, and number of vacuoles observed within *EM-P*. Cells in these images were stained with membrane stain, FM^{TM}5-95 (white), and DNA stain, PicoGreen^{TM} (red). Scale bars: 5µm.

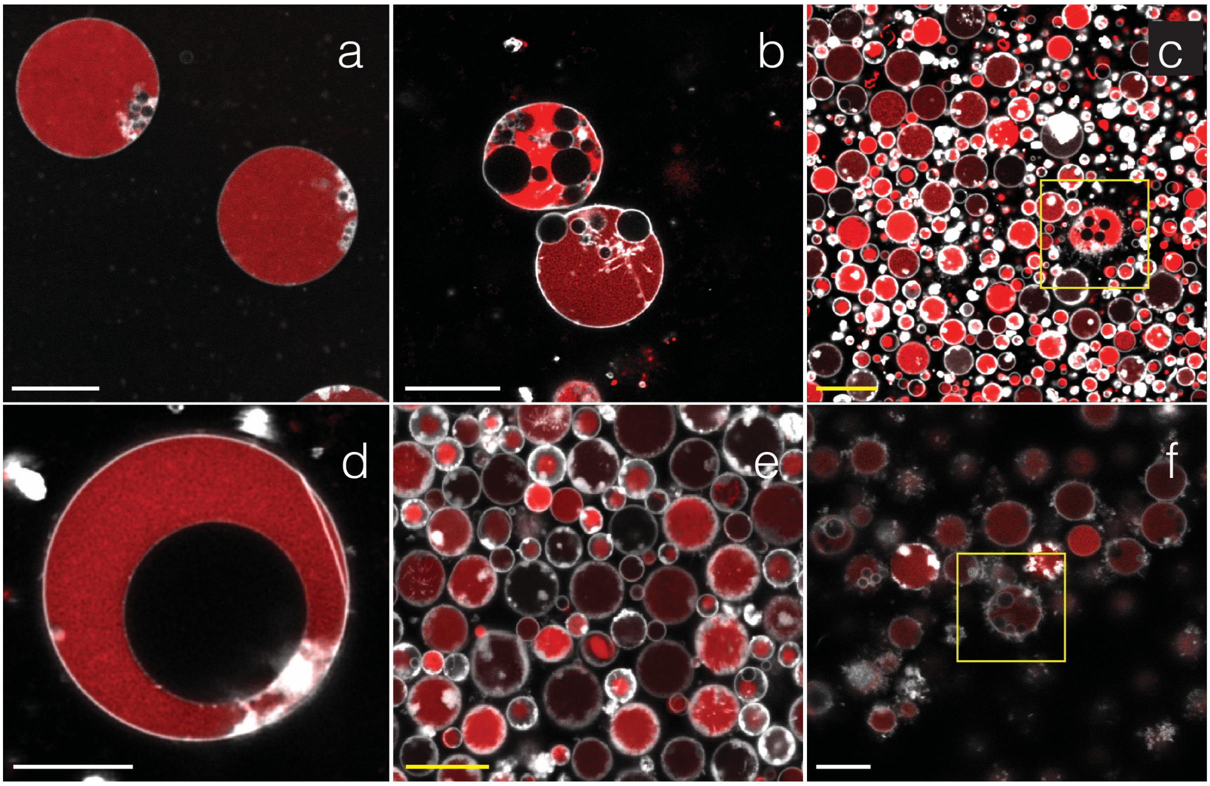

Figure S2: Morphological comparison of cells grown in the presence and absence of 3-methoxy benzamide. Images a-f are STED microscope images of *EM-P* grown in the presence (a-c) and absence (d-f) of 5 mM 3-methoxy benzamide. No significant differences were observed in the morphology or reproduction between these two incubations. Cells in these incubations developed intracellular vesicles and filamentous extensions (highlighted regions in images c & f). Cells in these images were stained with universal membrane stain, FM^{TM}5-95 (white), and DNA stain, PicoGreen^{TM} (red). Scale bars: 10μm.

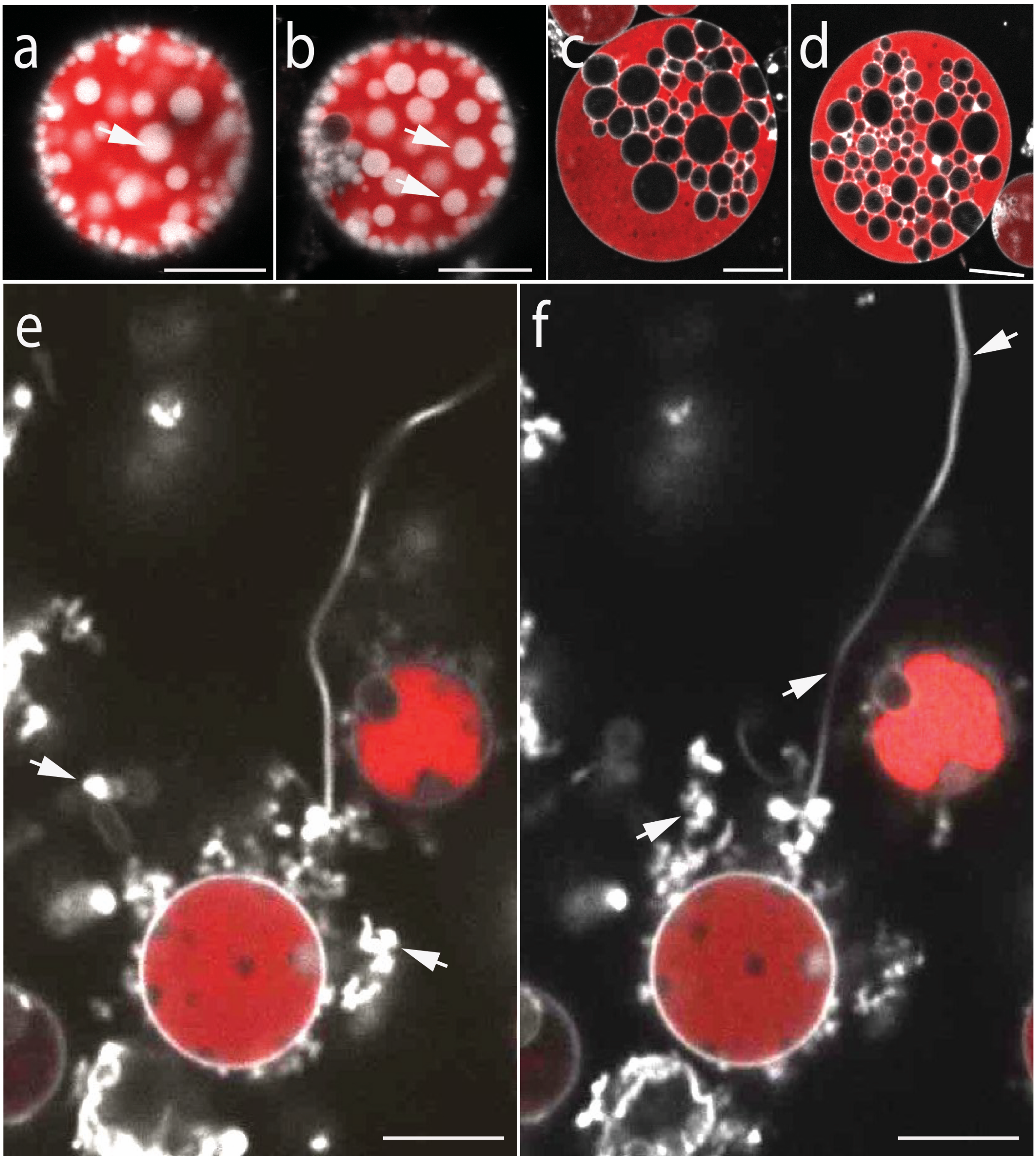

Figure S3: ***EM-P*** **cells in different growth stages exhibiting excess cell membrane.** Images a-f are the STED (a-d) and spinning disk confocal (e&f) microscope images of *EM-P* cells. Cells in these images were stained with universal membrane stain, FM^{TM}5-95 (white), and DNA stain, PicoGreen^{TM} (red). All cells exhibit excess membrane in the form of lipid globules (a & b, arrows), hollow intracellular vesicles, and filamentous extensions (e & f, arrows). Scale bars: 10µm.

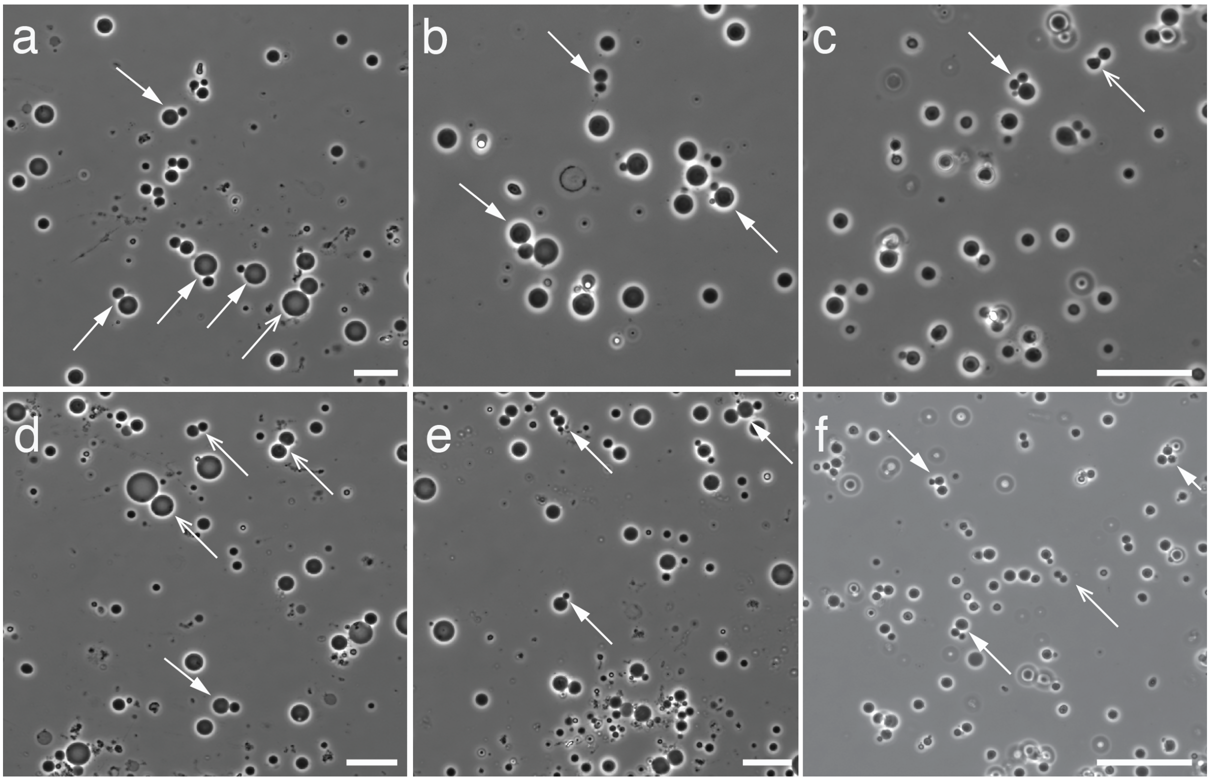

Figure S4: late log-phase *EM-P* cells undergoing reproduction. Images a-f show phase contrast images of log-phase *EM-P* cells that appear to reproduce by binary fission (open arrows) or budding (closed arrows). Scale bars: 10µm.

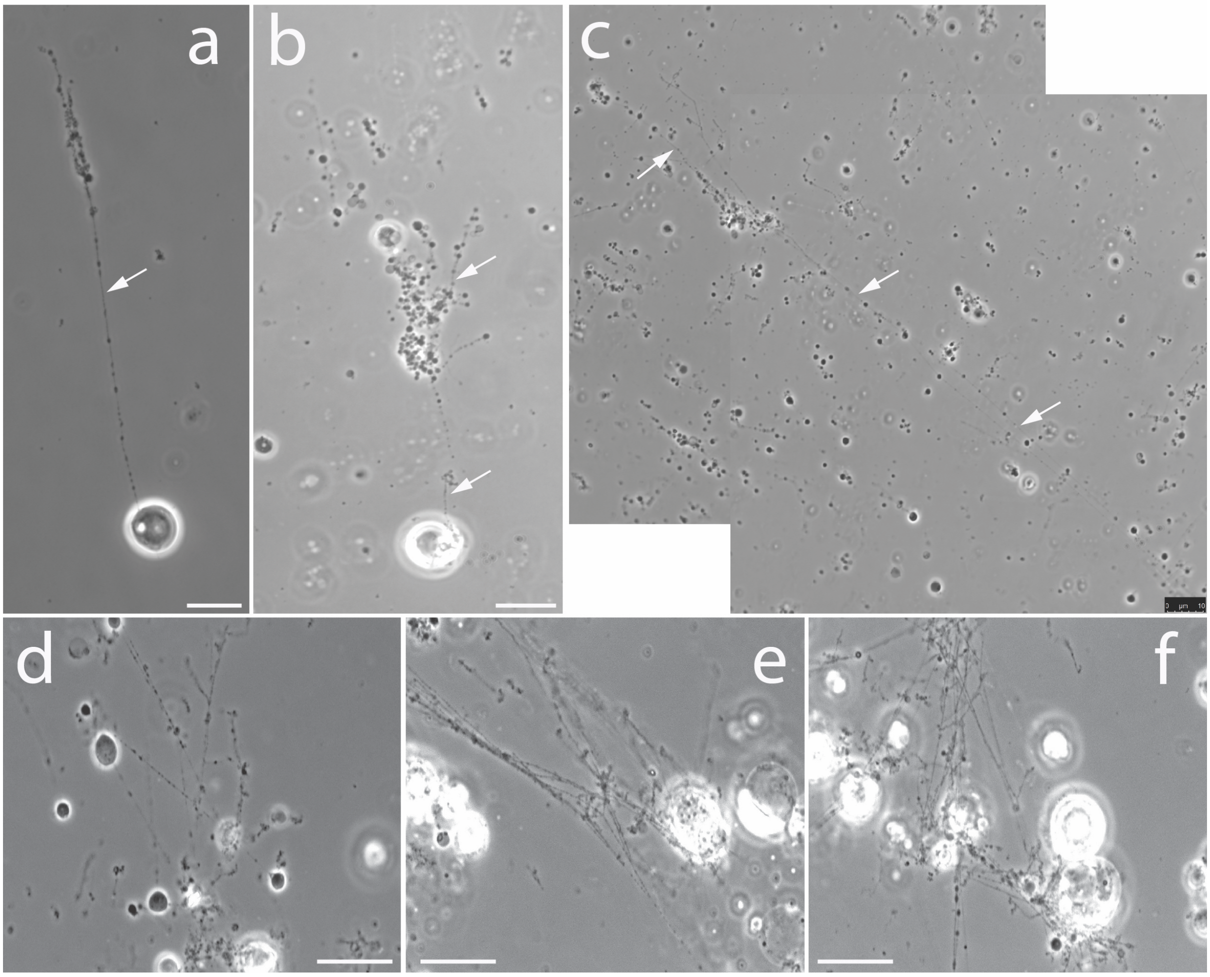

Figure S5: Influence of sudden increase in media osmolarity on *EM-P*'s morphology. Images a-f show *EM-P* cells that were transferred from 7%DSS-TSB to 15%DSS-TSB. Compared to the control incubations (like *EM-P* cells in Fig. S1), the sizes of the cells in 15%DSS-TSB were significantly smaller and contained longer filamentous extensions. Rather than being hollow, most filamentous extensions seem to have cytoplasm or spherical daughter cells (a-c, arrows). Scale bar: 10µm.

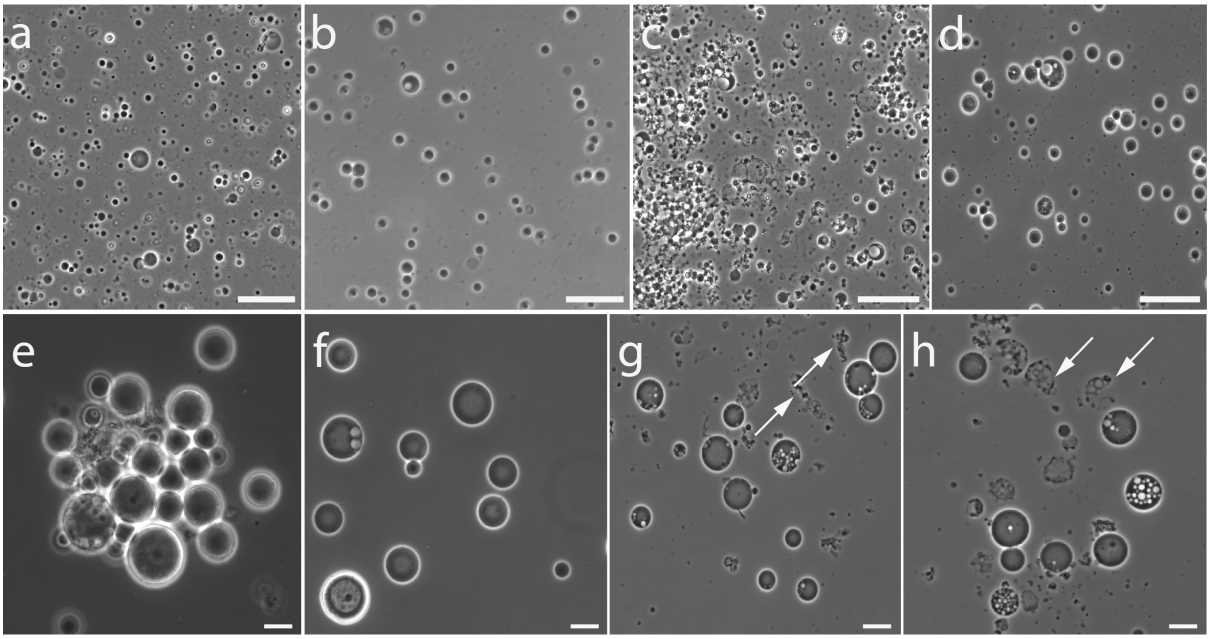

Figure S6: Influence of sudden decrease in media osmolarity on *EM-P*'s morphology. Images a-d show late log-phase *EM-P* cells in 7%DSS-TSB. Images e-h show *EM-P* cells that were transferred from 7%DSS-TSB to 4%DSS-TSB. Compared to the control incubations (a-d), the sizes of the cells in 4%DSS-TSB were significantly larger. We also noticed a lysis of cells after the transfer, possibly due to the sudden changes in the osmolarity. Arrows in images g & h, point to the cells that underwent lysis. Scale bar: 10µm.

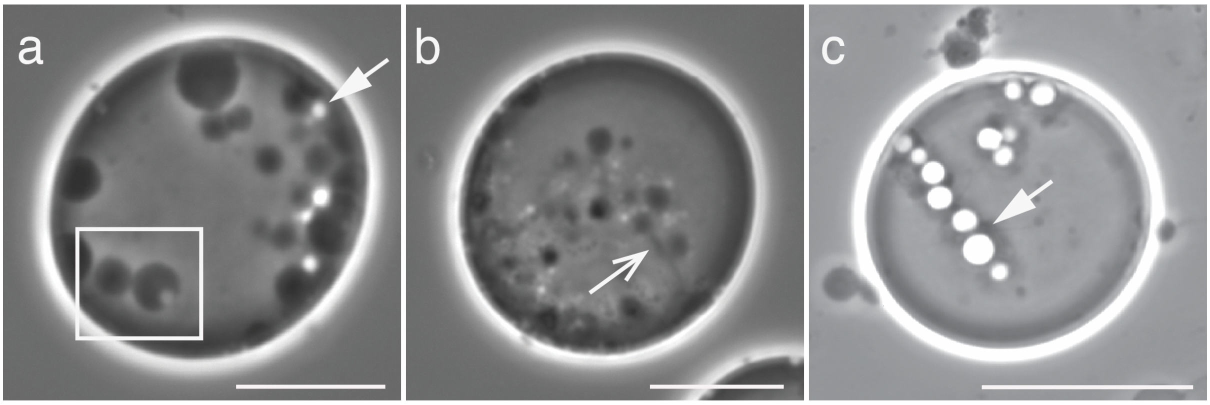

Figure S7: Transformation of lipid droplets into intracellular vesicles. Images a-c show different stages involved in the formation of intracellular vesicles *EM-P*. Image a shows an *EM-P* cell with a tiny hollow intracellular vesicle. The arrow in image-b points to an intracellular vesicle attached to the cell membrane by a tether. Image c shows the expansion of these membrane tethers into a string of hollow vesicles. Scale bars: 10µm.

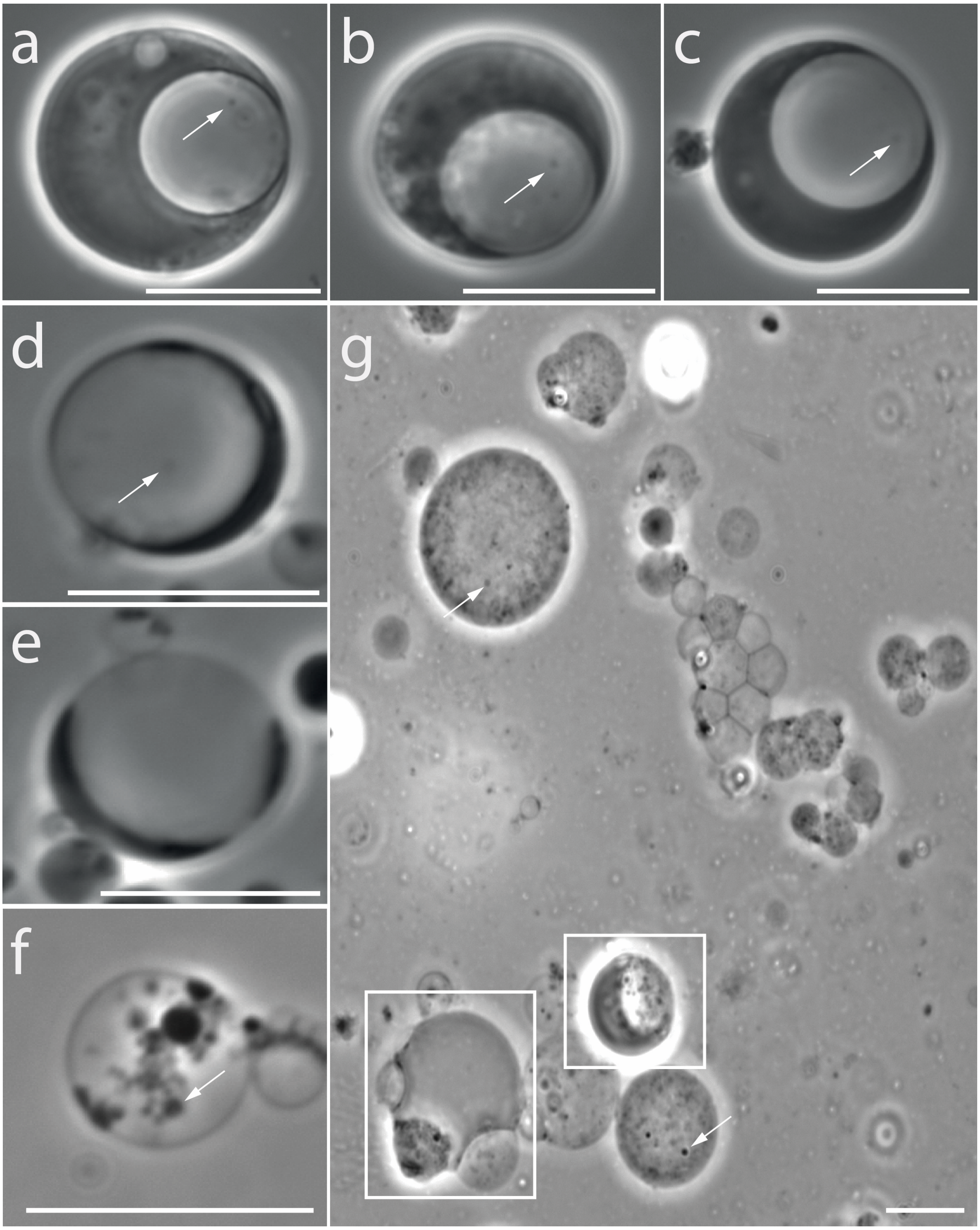

Figure S8: Morphological transformation of *EM-P* reproducing via forming internal daughter cells. Images a-g show phase-contrast images of *EM-P* reproducing by forming internal daughter cells. Image a-c shows *EM-P* cells with intracellular vesicles. White arrows in these images highlight barely visible daughter cells (more clearly visible in images f, g,

video S14 and S15). Images b-e show gradual depletion in the cytoplasmic content of the cell due to the loss of cytoplasm to daughter cells. Image-f shows an *EM-P* cell completely depleted of its cytoplasmic content to daughter cells. Image-g showed *EM-P* cells in their last growth stage when most cells transformed into spherical vesicles with tiny daughter cells (arrow) and some cells with relatively little cytoplasm (Boxed cells). Lysis and release of these daughter cells are shown in Figure 6h, & Video 16. Scale bars: 10μm.

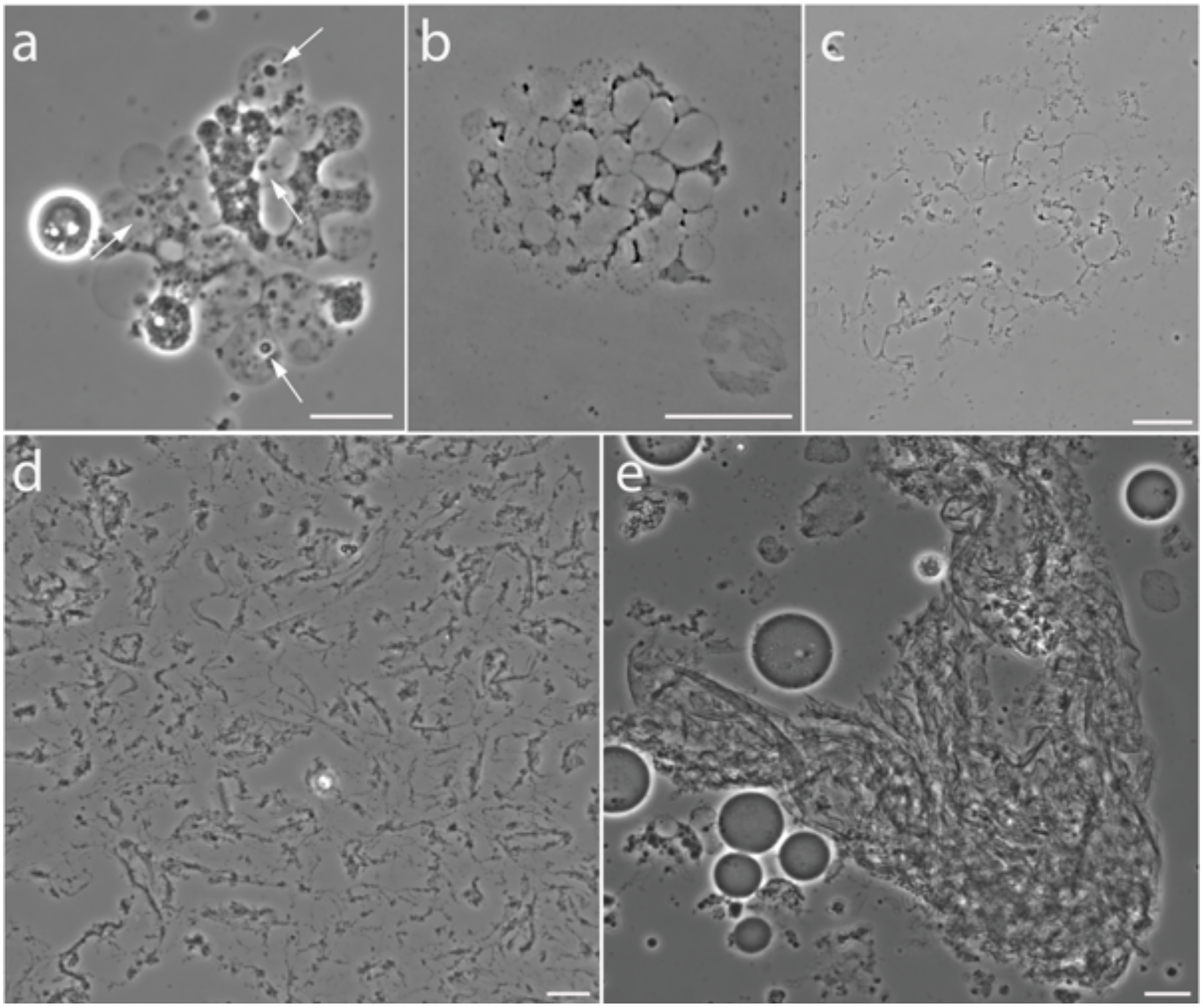

Figure S9: Membrane debris observed during the stationary growth phase.

Images a-e show sequential stages of membrane debris transformation. Image-a shows intracellular vesicles released by cell lysis of *EM-P* cells (Video 15-18). Arrows in image-a

point to tiny spherical daughter cells within these vesicles. Image b shows vesicles that underwent lysis to release daughter cells. Image c shows the leftover membrane debris after the release of daughter cells. Images d & e show subsequent aggregation and transformation of membrane debris into fabric-like structures. Scale bars: 10µm.

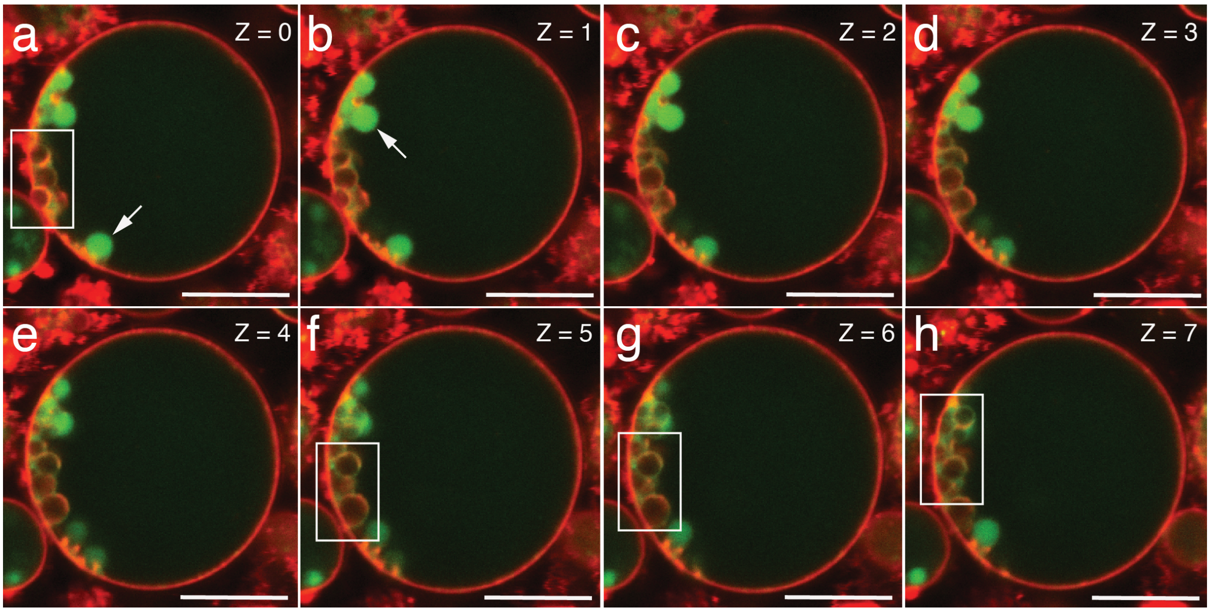

Figure S10: Transformation of L_d membrane into intracellular vesicles

Series of optical sections through late-log phase *EM-P* cells (0.2µm depth intervals). The cells were stained with universal membrane stain, FM™5-95 (all membrane, red), and *FAST*™ DiI (L_d-membrane specific, green). Scale bar: 10µm.

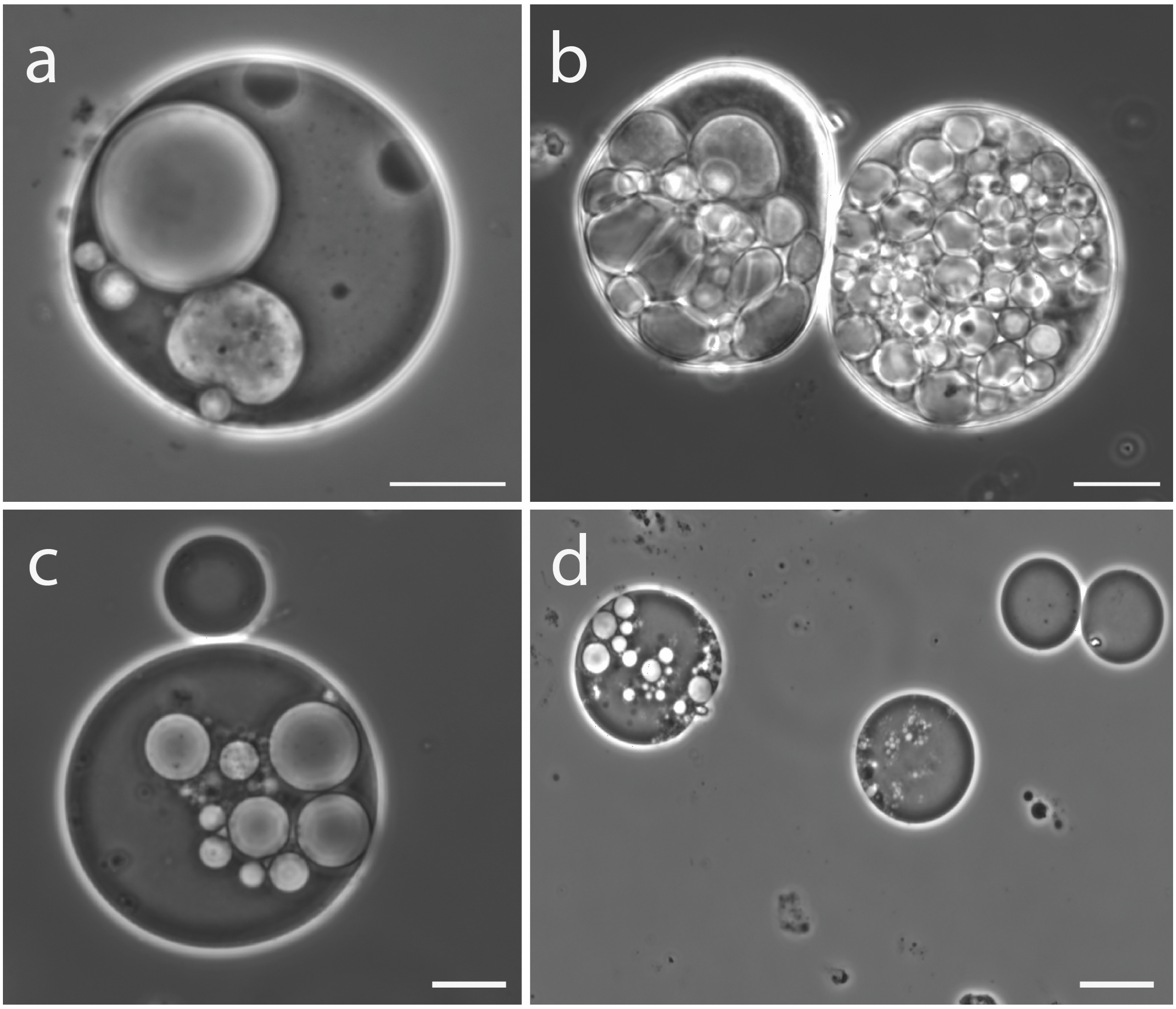

Fig. S11: *EM-P* cells reproducing via external daughter cells in 7%KCl-TSB

Images a-d show the sequential morphological transformation of *EM-P* when grown in the presence of 7% KCl-TSB. Scale bars: 10µm.

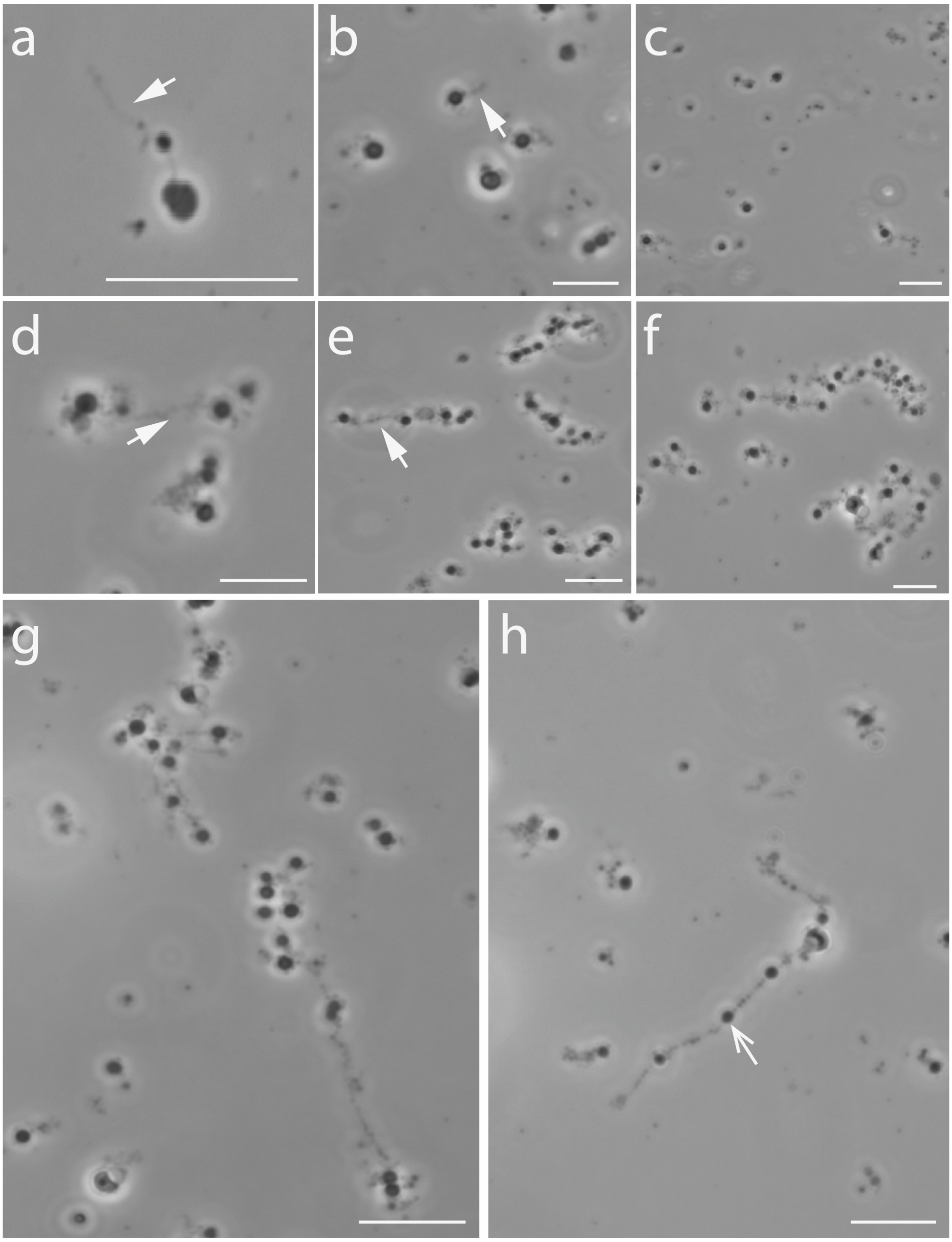

Figure S12: *EM-P* cells reproducing via external daughter cells in TSB-5%$MgCl_2$. Images a-h show the sequential morphological transformation of *EM-P* when grown in the presence of 5% $MgCl_2$-TSB. Closed arrows point to membrane tethers connecting individual daughter cells. Open arrows in these images point to spherical daughter cells within the filaments (videos S18 & S19). Scale bars: 10µm.

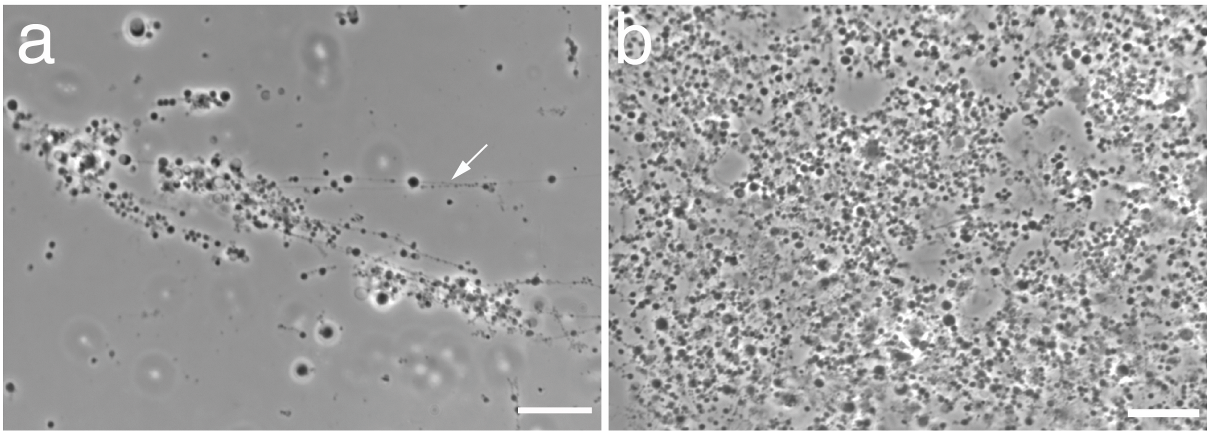

Figure S13: *EM-P* cells reproducing via external daughter cells in TSB-5%$MgCl_2$.

Images a & b show the late growth stage of *EM-P* when grown in the presence of 5% $MgCl_2$-TSB. Closed arrows point to membrane tethers connecting individual daughter cells (videos S18 & S19). Scale bars: 10µm.

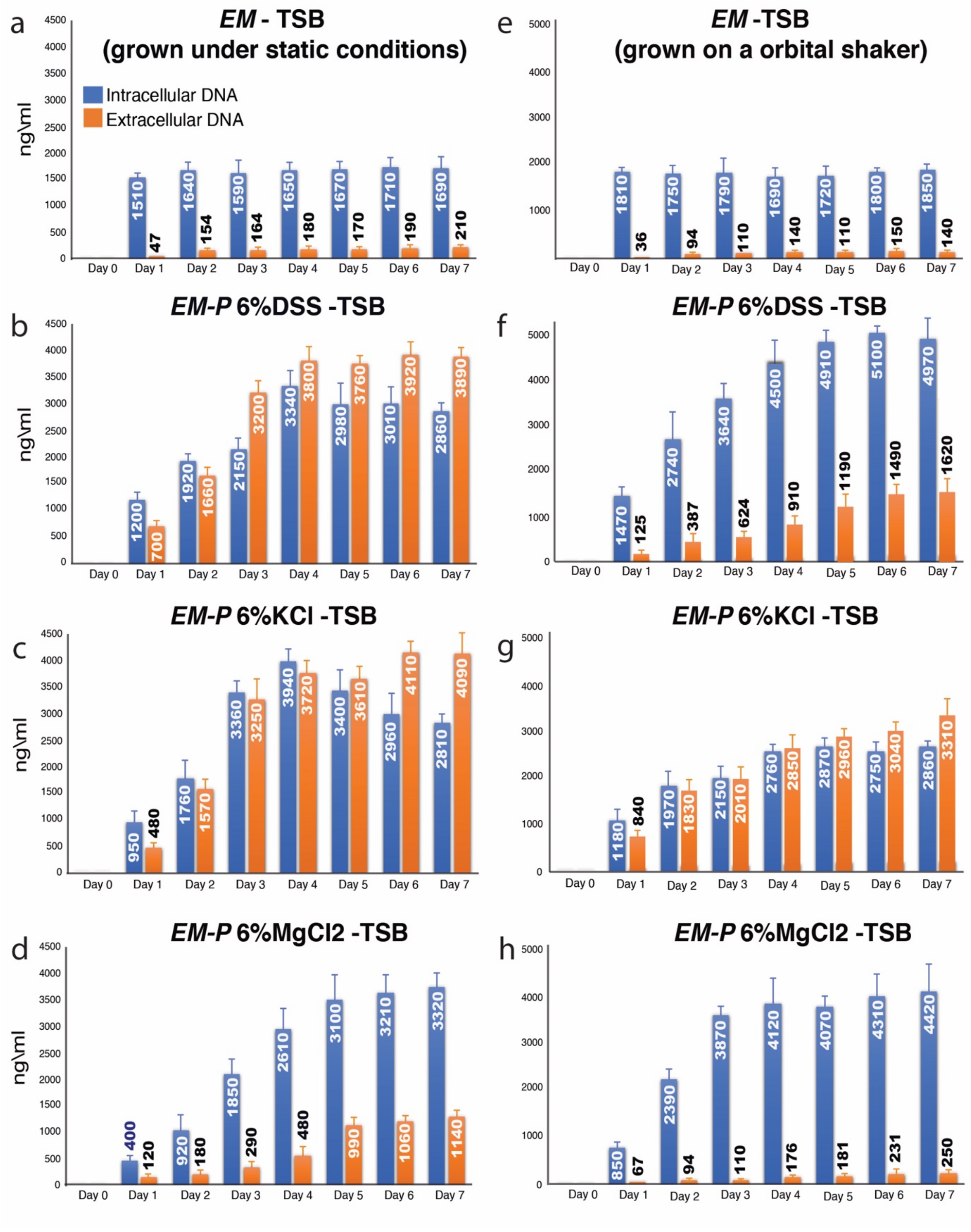

Figure S14: Reproductive efficiency of *EM-P*.

Plots a-h show the amount of intracellular and extracellular DNA in *EM-P* incubations during different growth stages and under different growth conditions. The plots (a-d) are the incubations done under static conditions. lot a shows the results of wild-type *EM*

(*Exiguobacterium strain M* with a cell wall). The rest of the plots b-d show *EM-P* grown in media containing DSS, KCl, and $MgCl_2$. The plots e-h are the incubations done on an orbital shaker. Plot-e shows the results of wild-type *EM* (*Exiguobacterium strain M* with a cell wall). The rest of the plots f-h show *EM-P* grown in media containing DSS, KCl, and $MgCl_2$. All experiments are biological repetitions from different batches of *EM-P*'s inoculum (n=5).

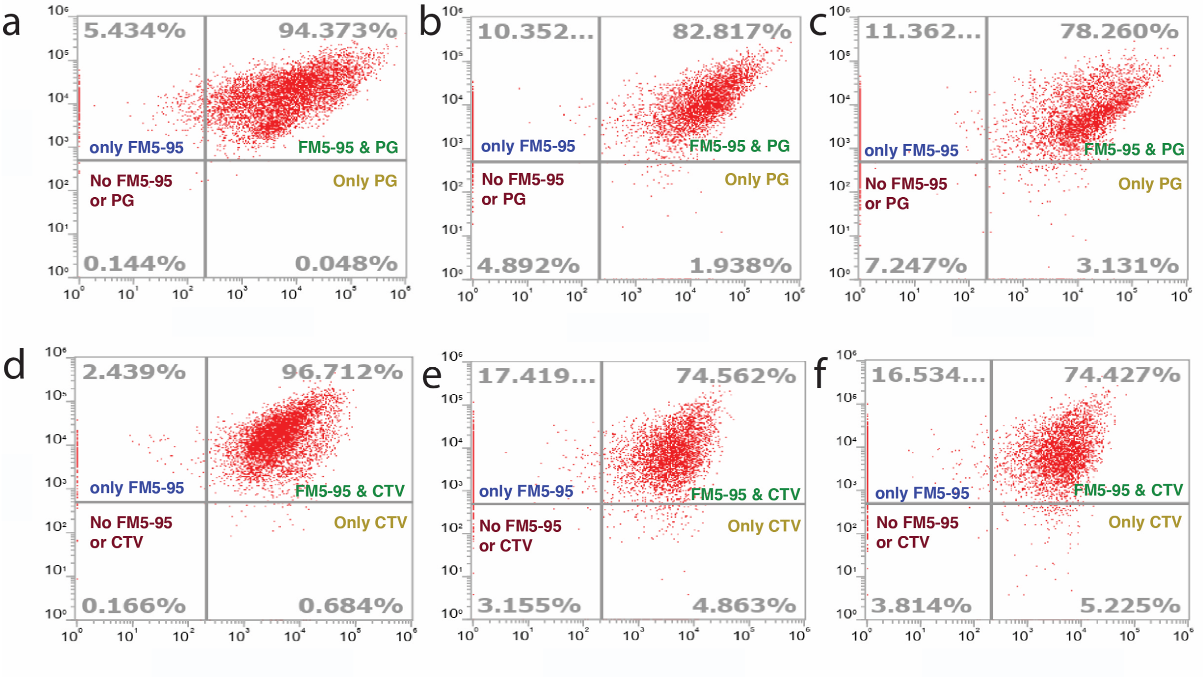

Figure S15: Viability of daughter *EM-P* daughter cells.

Figures a-f show flow cytometry quantification of different *EM-P* daughter cells. Cells from the stationary growth phase were passed through a 0.22µm filter to separate them from large parent cells and membrane debris (Fig. S9). Subsequently, one set of these cells were stained with FM5-95 (FM5, membrane) and Pico Green (PG, DNA), to determine if daughter cells had an intact membrane and received genetic material from the parent cell. The second set of cells were stained with membrane dye FM5-95 (FM5, membrane) and Cell Trace Violet (CTV, intracellular esterase) to determine if the daughter cells had an intact membrane and

exhibited cytoplasmic activity. A large fraction of cells in the top-right quadrant (FM5 & PG and FM5 & CTV) suggests most daughter cells are alive. Confocal images of these cells are shown in Fig. S16.

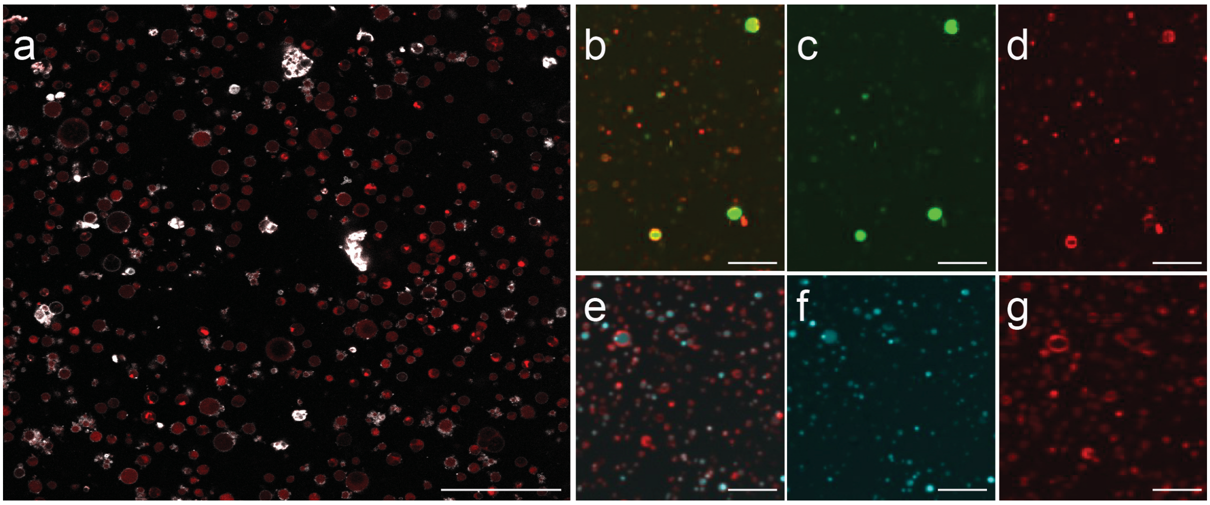

Fig. S16: *EM-P* cells in late stationery before and after filtration.

Image a shows a confocal microscope image of *EM-P* during the late growth stage. Cells in this image are stained with FM^{TM}5-95 (membrane, white) and PicoGreen^{TM} (DNA, red). Images b & e show *EM-P* daughter cells after passing through a 0.45µm filter to separate them from larger parent cells and membrane debris. Cells in images b-d are stained with FM5^{TM}-95 (cell membrane, red) and PicoGreen^{TM} (DNA, green). Images c & d show the same field of view in different channels for different dyes. Images e-g are images of *EM-P* daughter cells stained with FM5^{TM}-95 (cell membrane-red) and Cell Trace Violet^{TM} (cytoplasm, cyan). Images f & g show the same field of view in different channels specific for different dyes. Scale bar: 10 µm (a) and 2µm (b-g).

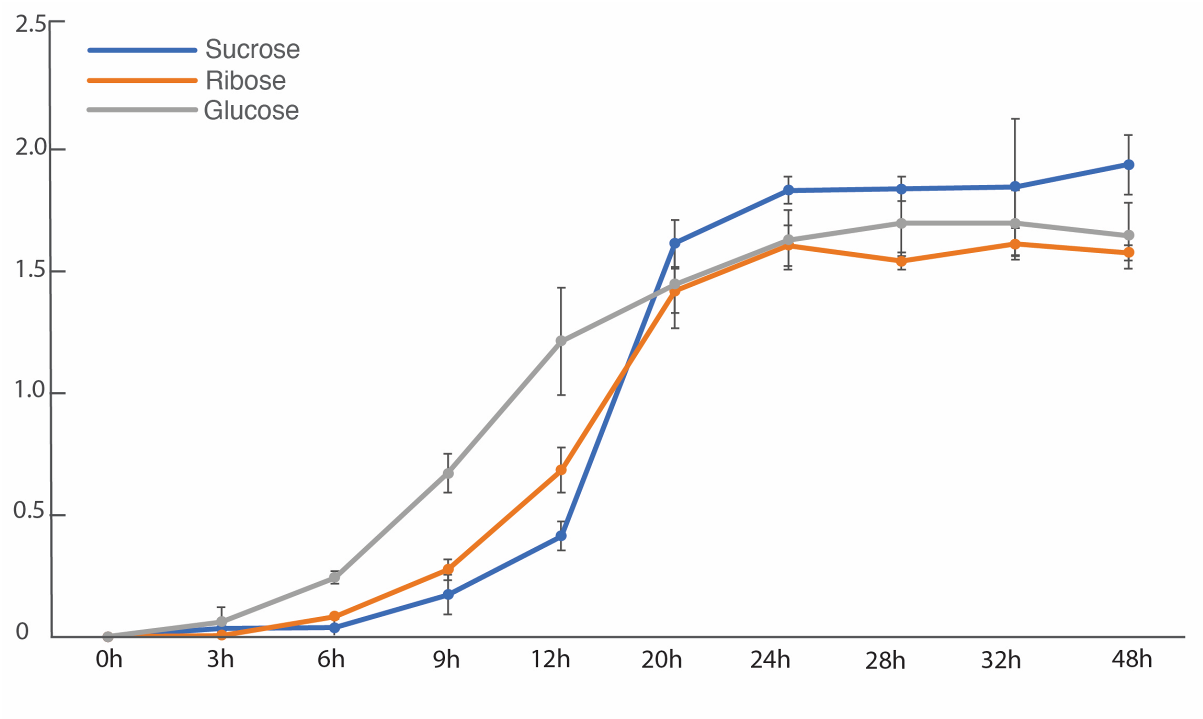

Fig. S17: Growth of EM-P in minimal salt media with a single carbon source:

The plot shows the growth characteristics of *EM-P* daughter cells in minimal salt media with glucose, ribose, or sucrose as the sole carbon sources. The X and Y-axis in the plot indicate the time in hours (h) and optical density (OD).

Supplementary videos:

Video S1: Log phase *EM-P* cells: Cells in this growth stage are spherical, and an order of magnitude larger than the inoculated cells. Most cells were devoid of hollow intracellular vesicles or filamentous extensions. Cells were never static and exhibited continuous surface undulations (large central *EM-P* cell). Cells with surface undulations can also be seen in videos S2 & S3. Scale bar: 5µm.

Video S2: Log phase *EM-P* cell: Cells within different stages of binary fission can be seen in this movie – uniformly spherical cells, ovoid cells (in the center of the frame), and cells segregated into two daughter cells (top left of the frame). Like the cells in video S1, most cells in this growth stage showed surface undulations. Scale bar: 5μm.

Video S3: Log phase *EM-P* cell undergoing binary fission: *EM-P* cells undergoing binary fission but still connected by narrow membrane tethers can be seen in the top left of the video. The bottom half of the video shows spherical cells with an unstable surface in a constant state of movement (surface undulations). Scale bar: 5μm.

Video S4 & S5: Sequential stages in forming external filamentous extensions: Video S4 shows a late log phase *EM-P* cell with filamentous and hollow spherical bud-like structure originating from the cell surface. Video S5 shows a gradual increase in the number of extensions originating from a single cell and the length of such filamentous extensions. Scale bar: 5μm.

Videos S6 to S8: Sequential stages involved in forming external daughter cells:
These videos show sequential stages involved in the formation of external daughter cells. Videos S6 to S8 show a gradual increase in the length of filamentous extensions and the formation of daughter cells within these filamentous extensions, transforming them into "a string or beads" morphology. These strings of daughter cells were never observed to be static. They seem to be in a continuous state of motion. Scale bar: 5μm.

Video S9 - S11: The fragmentation of the strings of daughter cells into individual daughter cells: These movies show the constant movement of individual daughter cells

within the filaments. This push and pull motion of cells within a string could have led to the rupture of membrane tethers (video S9) connecting the strings of daughter cells to the parent *EM-P* cell. Videos. S10 shows the subsequent rupture of these strings of daughter cells, leading to the formation of individual daughter cells (video S11). Scale bar: 5μm.

Video S12: *EM-L* cell with an intracellular vesicle: The video shows an *EM-P* cell with a hollow intracellular vesicle. Like the external filamentous extensions, intracellular vesicles, too, were in constant movement (oscillation). Scale bar: 5μm.

Video S13 - S15: *EM-P* cell with intracellular daughter cells: Cells in these images were stained with FM™5-95 (membrane, cyan) and PicoGreen™ (DNA, red). Videos show daughter cells within the hollow intracellular vesicles. Cells in video S13 show daughter cells formed as a string (the cell in the center of the video and one on the top-left). The video S13 shows similar *EM-P* cells with either individual daughter cells (most cells) or a string of daughter cells (cell towards the left of the video). The video S15 shows the stationary growth phase cells that were entirely depleted of their cytoplasm and internal daughter cells. Scale bar: 10μm.

Videos S16: Release of intracellular daughter cells: The video shows the slow-motion rupture of the vesicle membrane and the release of internal daughter cells. Scale bar:5μm.

Video S17: *EM-P* cells stained with FM™5-95 (all membrane, red) and FAST DiI™ (L_d-membrane, green). Most L_d (green) membrane within these cells is localized to the interior of the cells in the form of hollow intracellular vesicles. In comparison, the membrane extending out of the cell largely lacks L_d membrane. Scale bar: 5μm.

Video S18 & S19: *EM-P* cells grown in 7%$MgCl_2$: Video S18 shows those grown in 7%$MgCl_2$-TSB. Video 21 shows the magnified region of video S19, showing the constant movement of daughter cells within the filaments. Scale bar: 5µm.

Video S20: Early growth stage *EM-P* cells. Cells in these images were stained with FM™5-95 (membrane, cyan) and PicoGreen™ (DNA, green). In most cells, DNA is in a constant state of movement. Scale bar: 5µm.

Video S21: The movie shows mid-growth stage *EM-P* cells, undergoing lysis and release of DNA. Cells in these images were stained with FM™5-95 (membrane, red) and PicoGreen™ (DNA, green). Scale bar: 5µm.

Video S22: *EM-P* cell lysis: The video shows late log phase *EM-P* cells, undergoing spontaneous lysis. Scale bar: 5µm.
